# A Synoptic Assessment of the Amazon River-Ocean Continuum during Boreal Autumn: From Physics to Plankton Communities and Carbon Flux

**DOI:** 10.3389/fmicb.2017.01358

**Published:** 2017-07-31

**Authors:** Moacyr Araujo, Carlos Noriega, Gbekpo Aubains Hounsou-gbo, Doris Veleda, Julia Araujo, Leonardo Bruto, Fernando Feitosa, Manuel Flores-Montes, Nathalie Lefèvre, Pedro Melo, Amanda Otsuka, Keyla Travassos, Ralf Schwamborn, Sigrid Neumann-Leitão

**Affiliations:** ^1^Department of Oceanography (DOCEAN), Federal University of Pernambuco (UFPE) Recife, Brazil; ^2^Brazilian Research Network on Global Climate Change (Rede CLIMA) São José dos Campos, Brazil; ^3^International Chair in Mathematical Physics and Applications (UNESCO Chair), Université d'Abomey-Calavi Cotonou, Benin; ^4^IIRD-LOCEAN (Laboratoire d'Océanographie et du Climat: Expérimentations et Approches Numériques), Sorbonne Universités (Université Pierre et Marie Curie, Centre National de la Recherche Scientifique, Muséum National d'Histoire Naturelle) Paris, France

**Keywords:** Amazon River-Ocean Continuum, biogeochemistry, carbon cycle, plankton communities, Camadas Finas III, tropical Atlantic

## Abstract

The Amazon generates the world's largest offshore river plume, which covers extensive areas of the tropical Atlantic. The data and samples in this study were obtained during the oceanographic cruise Camadas Finas III in October 2012 along the Amazon River-Ocean Continuum (AROC). The cruise occurred during boreal autumn, when the river plume reaches its maximum eastward extent. In this study, we examine the links between physics, biogeochemistry and plankton community structure along the AROC. Hydrographic results showed very different conditions, ranging from shallow well-mixed coastal waters to offshore areas, where low salinity Amazonian waters mix with open ocean waters. Nutrients, mainly ${\text{NO}}_{3}^{-}$ and ${\text{SiO}}_{2}^{-}$, were highly depleted in coastal regions, and the magnitude of primary production was greater than that of respiration (negative apparent oxygen utilization). In terms of phytoplankton groups, diatoms dominated the region from the river mouth to the edge of the area affected by the North Brazil Current (NBC) retroflection (with chlorophyll *a* concentrations ranging from 0.02 to 0.94 mg m^−3^). The North Equatorial Counter Current (NECC) region, east of retroflection, is fully oligotrophic and the most representative groups are Cyanobacteria and dinoflagellates. Additionally, in this region, blooms of cyanophyte species were associated with diatoms and Mesozooplankton (copepods). A total of 178 zooplankton taxa were observed in this area, with Copepoda being the most diverse and abundant group. Two different zooplankton communities were identified: a low-diversity, high-abundance coastal community and a high-diversity, low-abundance oceanic community offshore. The CO_2_ fugacity (fCO_2_sw), calculated from total alkalinity (1,450 < TA < 2,394 μmol kg^−1^) and dissolved inorganic carbon (1,303 < DIC < 2,062 μmol kg^−1^) measurements, confirms that the Amazon River plume is a sink of atmospheric CO_2_ in areas with salinities <35 psu, whereas, in regions with salinities >35 and higher-intensity winds, the CO_2_ flux is reversed. Lower fCO_2_sw values were observed in the NECC area. The ΔfCO_2_ in this region was less than 5 μatm (−0.3 mmol m^−2^ d^−1^), while the ΔfCO_2_ in the coastal region was approximately 50 μatm (+3.7 mmol m^−2^ d^−1^). During the cruise, heterotrophic and autotrophic processes were observed and are indicative of the influences of terrestrial material and biological activity, respectively.

## Introduction

Each late summer/autumn, the Amazon River plume covers ~2 × 10^6^ km^2^ of the western tropical North Atlantic Ocean (WTNA) (DeMaster and Pope, 1996; Smith and Demaster, 1996; Ternon et al., 2000; Körtzinger, 2003; Cooley et al., 2007). The Amazon River has the greatest discharge of any global river and accounts for ~20% of all of the riverine input to the oceans, more than the next seven largest rivers combined. The mean discharge of the Amazon River is approximately 150,000 m^3^ s^−1^ and is responsible for approximately half of all the freshwater input into the tropical Atlantic (Baumgartner and Reichel, 1975; Yoo and Carton, 1990; Carton, 1991). This rate varies by 50% between a maximum in May–June and a minimum in November–December (Richey et al., 1989; Carton, 1991). The impacts of this plume on the WTNA include nutrients, microorganisms, and fresh water fluxes that contribute to enhanced biological activity and carbon sequestration over a million square kilometers of tropical ocean.

The Amazon River-Ocean Continuum (AROC) is an energetic region subjected to strong geophysical forcing's, including the Amazon River discharge, the North Brazil Current-North Equatorial Counter Current (NBC-NECC) system, macrotides and strong trade winds (Silva et al., 2005, 2009, 2010). The dynamics of the WTNA are also affected by the seasonal transposition of the Intertropical Convergence Zone (ITCZ). This region is also known as also an important location of heat exchange through a complicated system of currents and water masses around the equator (Stramma and Schott, 1999).

On the western edge of the WTNA, the northward migration of the ITCZ results in the retroflection of the NBC, which feeds the NECC with eastward-transported waters of the Amazon River plume (Richardson and Reverdin, 1987; Fonseca et al., 2004; Coles et al., 2013). The waters from the Amazon River and the increased rainfall caused by the presence of the ITCZ are the major sources of fresh water along the western edge of the WTNA.

These contributions are sensitive to the quantity and composition of the river discharge itself. The upper Amazon River is a source of CO_2_ to the atmosphere (210 ± 60 Tg C year^−1^; Richey et al., 2002), supported by organic matter mineralization and carbon dioxide and organic matter export from flooded wetlands (Abril et al., 2014). When transported through the salt gradient, the high concentrations of nutrients in the river water are diluted via mixing with ocean water, favoring the growth of primary producers and decreasing the amount of associated organic carbon (Chen et al., 2012). The spread of Amazon waters in the tropical Atlantic is also known to support significant N_2_ fixation through diatom-diazotroph associations, which represents the main carbon sequestration pathway within the plume (Subramaniam et al., 2008; Yeung et al., 2012).

The different factors that contribute to CO_2_ undersaturation in the Amazon River plume and its seasonal variability remain poorly understood. Ternon et al. (2000) estimated that primary production within the plume could be responsible for approximately 30% of the observed CO_2_ undersaturation. Additionally, Cooley et al. (2007) suggested that net primary production in the river plume would enhance the observed CO_2_ undersaturation by a hundredfold. Elucidation of the physical and biological processes responsible for the modulation of the sea surface carbon dioxide fugacity (fCO_2_) in the Amazon River plume and the WTNA may help better constrain the role of the tropical Atlantic in the global sea-air CO_2_ exchange.

The main objective of this work is to improve our understanding of the processes and organisms responsible for carbon and nutrient cycling along a large-scale tropical river-ocean continuum (Amazon River to offshore), focusing on the nearshore and offshore WTNA. This combined river-ocean continuum represents one of the largest environmental gradients on land and in the ocean in the world and stretches across thousands of km from the continental shelf to the middle of the Atlantic.

## Materials and methods

### Hydrography and currents

The dispersal of Amazon River water forms a brackish water plume that can exceed 10^6^ km^2^, reaching latitudes as far from the river mouth as 30°W (Coles et al., 2013) or even 25°W when the North Equatorial Countercurrent (NECC) is strong (Lefèvre et al., 1998). Thus, the large areas of fresh sea surface waters (≤33 salinity) observed in the region are primarily due to the Amazon discharge.

The data and samples in this study were obtained during the oceanographic cruise Camadas Finas III (hereafter, CF3) aboard the research vessel *NHo. Cruzeiro do Sul – H38* (DHN/Brazilian Navy). This cruise was performed during October 9th–31st, 2012, corresponding to the period when most of the Amazon plume is transported eastward and coinciding with the northernmost annual position of the Intertropical Convergence Zone (ITCZ).

**Figure 1:**
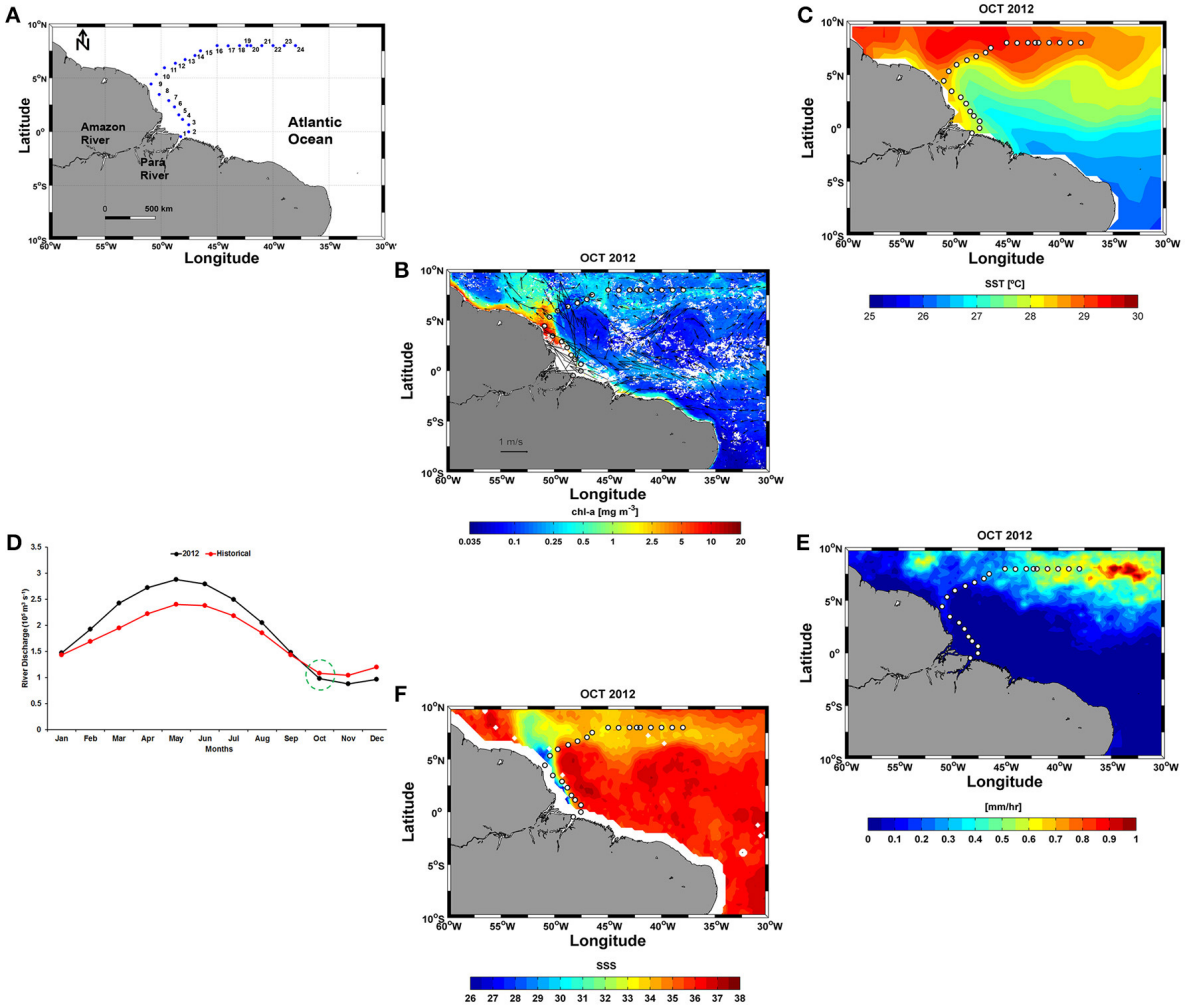
**(A)** Positions of the 24 stations sampled along the ship track during the Camadas Finas III (CF3) cruise; **(B)** Sea surface currents (cm s^−1^) and Chl-a (mg m^−3^) in October 2012; **(C)** SST for the same period, obtained from Objectively Analyzed air-sea Fluxes (OAflux); **(D)** Monthly Amazon River discharge (m^3^ s^−1^) in October 2012 and climatology (1982–2012); **(E)** Monthly precipitation data sets (mm h^−1^); and **(F)** SSS for October 2012 derived from the Soil Moisture and Ocean Salinity (SMOS).

The ship track encompassed the outer estuary portion, the alongshore northwestern NBC region, the NBC retroflection area and the eastward NECC plume transport to 38°W (Figure 1A).

In this work, monthly sea surface currents (cm s^−1^) in October 2012 were obtained from the Geostrophic and Ekman Current Observatory (GEKCO) and downloaded from the Center for Topographic studies of the Ocean and Hydrosphere (CTOH) (http://ctoh.legos.obs-mip.fr/products/global-surface-currents, 1/4° resolution) (Figure 1B). The current vectors in Figure 1B were superimposed on a chlorophyll *a* (Chl-*a*) distribution map (SeaWiFS, https://podaac.jpl.nasa.gov/dataset/SeaWiFS_L3_CHLA_Monthly_9km_, 1/12° resolution) for the same period. Sea surface temperature (SST) data were obtained from the Objectively Analyzed air-sea Fluxes (OAflux) project (http://oaflux.whoi.edu/, 1° resolution) (Figure 1C). Monthly Amazon River discharges (m^3^ s^−1^) were obtained from the National Water Agency (ANA) in the Amazon basin (http://www2.ana.gov.br/Paginas/EN/default.aspx). The seasonal evolution of the river discharge in 2012 did not show was not significantly different (*t*-test; *p*: 0.49; α: 0.05) from that of historical climatological series (1982–2012) (Figure 1D). Monthly precipitation data (mm h^−1^) was obtained from the Tropical Rainfall Measuring Mission (TRMM) (Huffman et al., 2007) (http://precip.gsfc.nasa.gov/, 1/4° resolution) (Figure 1E). Sea surface salinity (SSS) data (1/4° resolution) derived from the Soil Moisture and Ocean Salinity (SMOS) mission were obtained from the Ocean Salinity Expertise Center (CECOS) of the Centre National d'Etudes Spatiales- Institut Français de Recherche pour l'Exploitation de la Mer (IFREMER), Centre Aval de Traitemenent des Données (CATDS), France (Figure 1F).

### Chemical analysis, apparent oxygen utilization (AOU) and N^*^-DINxs indices

Dissolved oxygen (DO) was determined using the modified Winkler method according to Strickland and Parsons (1972) with an accuracy of ±1.3 μmol L^−1^. The apparent oxygen utilization (AOU) represents one estimate of the O_2_ utilized by biochemical processes relative to a preset value. AOU (mL L^−1^) is calculated as the difference between the O_2_ gas solubility (${\text{O}}_{2}^{*}$) and the measured O_2_ concentration and is expressed as follows:

(1) $$\text{AOU}=\left({{\text{O}}_{2}}^{*}\right)-\left({\text{O}}_{2}\right)$$

where ${\text{O}}_{2}^{*}$ is calculated as a function of *in situ* temperature and salinity at one atmosphere of total pressure. The ${\text{O}}_{2}^{*}$ values were calculated using the equation of Garcia and Gordon (1992) based on the ${\text{O}}_{2}^{*}$ values of Benson and Krause (1984). Additionally, O_2_ is the measured O_2_ concentration (mL L^−1^). Dissolved inorganic nutrients (ammonia (NH_3_+${\text{NH}}_{4}^{+}$) + nitrite (${\text{SiO}}_{2}^{-}$) + nitrate (${\text{NO}}_{3}^{-}$), phosphate (${\text{PO}}_{4}^{-}$), and reactive silicate (${\text{SiO}}_{2}^{-}$)) were analyzed according to Grasshoff et al. (1983). The precision was ±0.05 μmol for ${\text{NO}}_{3}^{-}$, ±0.02 μmol for ${\text{SiO}}_{2}^{-}$, ±0.10 μmol for ${\text{NH}}_{4}^{+}$, 0.01 μmol for ${\text{PO}}_{4}^{-}$, and 0.25 μmol for ${\text{SiO}}_{2}^{-}$. The accuracy was 2% for ${\text{PO}}_{4}^{-}$, 3% for ${\text{NO}}_{3}^{-}$ and ${\text{SiO}}_{2}^{-}$, 5% for ${\text{NH}}_{4}^{+}$, and 6% for ${\text{SIO}}_{2}^{-}$. Dissolved inorganic nitrogen (DIN) was calculated as the sum of ${\text{NO}}_{3}^{-}$ + ${\text{SiO}}_{2}^{-}$ + ${\text{NH}}_{4}^{+}$. The indices N^*^ (N^*^ = ${\text{NO}}_{3}^{-}$ - 16${\text{PO}}_{4}^{-}$ + 2.90; Gruber and Sarmiento, 1997; Deutsch et al., 2001;) and DINxs (DINxs = ${\text{NO}}_{3}^{-}$ - 16${\text{PO}}_{4}^{-}$; Bates and Hansell, 2004; Hansell et al., 2004) represent the relative abundances of nitrate and phosphate. These indices measure the departure from classical Redfield ratios of the dissolved inorganic forms of nitrogen and phosphorus (the N^*^ and DINxs indices differ only in the offset of 2.90 μmol L^−1^, a value that was intended to fix the global mean N^*^ value to zero). Negative values of DINxs (or N^*^ values < 2.9 μmol L^−1^) indicate a deficit in N relative to P with respect to the requirements for Redfieldian production of organic matter; positive values of DINxs (or N^*^ values >2.9 μmol L^−1^) indicate excess N relative to P. The values obtained for N^*^ are shown in **Table 2**.

### The CO_2_ system

Seawater samples were collected for total inorganic carbon (DIC) and total alkalinity (TA) analyses to assess the key parameters of the CO_2_ system. DIC and TA were measured via potentiometric titration using a closed cell, following the method of Edmond (1970). Equivalent points were calculated using the code published by (The Department of Energy, 1994). Certified reference material, supplied by Professor A. Dickson (Scripps Institutions of Oceanography, San Diego, USA), was used for calibration. The accuracy was estimated at 3 μmol kg^−1^.

The sea surface fCO_2_ was calculated from TA, DIC, temperature and salinity using the CO2calc®software (Robbins et al., 2010). The K_1_ and K_2_ dissociation constants of the carbonic acid used in the calculations were those obtained by Mehrbach et al. (1973) and refitted by Dickson and Millero (1987), and the sulfate dissociation constants were from Dickson (1990a,b).

The monthly averaged atmospheric CO_2_ mole fraction (*X*CO_2_atm, ppm) recorded at the NOAA/Earth System Research Laboratory (ESRL) Global Monitoring Division station closest to the Amazon River plume (Ragged Point, Barbados, 13.17°N, 59.43°W; http://www.esrl.noaa.gov/gmd/ccgg/iadv/) was used for the atmospheric fCO_2_ (fCO_2_atm) and sea-air CO_2_ flux calculations. The fCO_2_atm was calculated as follows:

(2) $${\text{fCO}}_{2}\text{atm}=X{\text{CO}}_{2}\text{atm}\times \left(\text{P -}p{\text{H}}_{2}\text{O}\right)\text{Cf}$$

where P is the atmospheric pressure (atm), *p*H_2_O is the water vapor pressure at 100% humidity (atm) calculated from SST and SSS, and Cf is the fugacity coefficient calculated according to Weiss (1974). The atmospheric pressure in October 2012 was obtained from the National Centers for Environmental Prediction (NCEP)/National Center for Atmospheric Research Reanalysis project, initially at a 2.5° resolution, and linearly interpolated to the scale of the working grid (1/4° resolution). The air-sea CO_2_ flux (CO_2_ fluxes, in mmol m^−2^ d^−1^) was then calculated as follows:

(3) $${\text{CO}}_{2}\text{fluxes}=k\times S_{o}\times \left({\text{fCO}}_{2}\text{sw}-{\text{fCO}}_{2}\text{atm}\right)$$

where *S*_*o*_ is the solubility of CO_2_ (mol kg^−1^ atm^−1^) as a function of SST and SSS (Weiss, 1974), *k* is the gas transfer velocity (m d^−1^), and fCO_2_sw is the fCO_2_ of the surface ocean waters (calculated from that measured along the CF3 tracks). The term *k* was calculated according to Sweeney et al. (2007):

(4) k=0.27U102(sc660)−0.5

where Sc is the Schmidt number and U_10_ is the wind speed (m d^−1^) at 10 m above sea level. U_10_ data for October 2012 were taken from Advanced Scatterometer (ASCAT) observations at 10 m (ftp://ftp.ifremer.fr/ifremer/cersat/products/gridded/MWF/L3/ASCAT/, 1/4°resolution).

A positive flux value represents net release of CO_2_ from the sea surface, and a negative flux value represents absorption of atmospheric CO_2_ by the sea.

### Plankton communities and Chl-*a*

Samples for the study of phytoplankton communities were taken using vertical tows with a plankton net with a 20 μm mesh, starting at 10 m below the recorded deep chlorophyll maximum (DCM) up to the surface. In total, 18 tows were performed. The samples were subsequently fixed with neutral formaldehyde solution to a final concentration of 4%, according to the methods of Newell and Newell (1963). To analyze the phytoplankton composition, the samples were gently mixed to homogeneously suspend the organisms, then 0.5 mL aliquots were qualitatively analyzed using an optical microscope with 100× and 400× magnification. The organisms were identified by consulting specialized literature. The international database Algaebase was used to classify and check the scientific names of the taxa (Guiry and Guiry, 2016). The relative abundances of the taxa were calculated as described by Lobo and Leighton (1986). The specific diversity of the phytoplankton was evaluated using the Shannon index (Shannon, 1948), and the evenness was calculated according to Pielou (1977) using PRIMER 6.0.

The method for determining the Chl-*a* concentration was the spectrophotometric analysis described in UNESCO (1966).

For the zooplankton sampling, a Bongo frame with four nets (with mesh sizes of 64-, 120-, 300-, and 500 μm) was used. For this study, samples from three nets with the following mesh sizes and diameters were analyzed: 64 μm/30 cm; 120 μm/30 cm, and 300 μm/60 cm. The Bongo net was hauled obliquely at a speed of 2–2.5 knots at depths between 15 m nearshore and from 200 m to the surface at stations beyond the shelf break. A flowmeter (Hydrobios, Kiel) was attached to the opening of each net. Additionally, plankton nets with a 200 μm mesh size were used for vertical hauls. At each station, two vertical hauls were conducted: one shallow vertical haul from the base of the mixed layer to the surface (Tropical Surface Water, TSW) and one deep vertical haul from the mid-thermocline to the surface (TSW and South Atlantic Central Water, TSW+SACW). In areas shallower than 70 m with no thermocline, only a single haul was conducted, from 5 m above the bottom to the surface. Neuston tows were also conducted at all stations. The neuston net used was a David-Hempel nautical aluminum catamaran manufactured by Hydro-Bios (Kiel, Germany). This equipment is composed of two superimposed nets, one at the air/water interface and another 7.5 cm below the interface. The two nets have rectangular mouths, each with a width of 30 cm and a height of 15 cm. The upper net samples 7.5 cm above the air-water interface to 7.5 cm below the interface. The lower net, equipped with a flowmeter (Hydro-Bios, Germany), samples the sub-surface layer (or hyponeuston), from 7.5 cm depth to 22.5 cm depth. The total mouth area is 0.066 m^2^ (0.022 m^2^ for the upper net and 0.044 m^2^ for the lower net). The sampling duration was approximately 20 min. A total of 291 samples were taken and preserved in a 4% buffered formalin-seawater solution, buffered with 0.5 g L^−1^ sodium tetraborate. The biomass was estimated via the wet-weight method (Omori and Ikeda, 1984).

### Statistical analyses

To test for differences between coastal and oceanic stations and between day and night, we used Mann-Whitney *U*-tests or Student's *t*-tests, depending on the normality and homoscedasticity of the data (Zar, 1996). Accordingly, Kruskal-Wallis ANOVA, linear regression analysis, and Pearson's correlation (Zar, 1996) were used to analyze the relationships between variables.

Cluster analysis was used to identify spatial divisions within the cruise track. Physical (σ-t), chemical (${\text{NO}}_{3}^{-}$ and DIC) and plankton (phytoplankton and zooplankton biomass) parameters were used for Hierarchical Agglomerative Cluster (CAH) analysis of Pearson's similarity and were agglomerated via the unweighted pair-group average method.

Principal components analysis (PCA) was performed to analyze the structure of the multivariate data using 27 key oceanographic parameters.

All statistical analyses were performed using the XLSTAT® 2010 software.

## Results

### Temperature, salinity, and density

**Figure 2:**
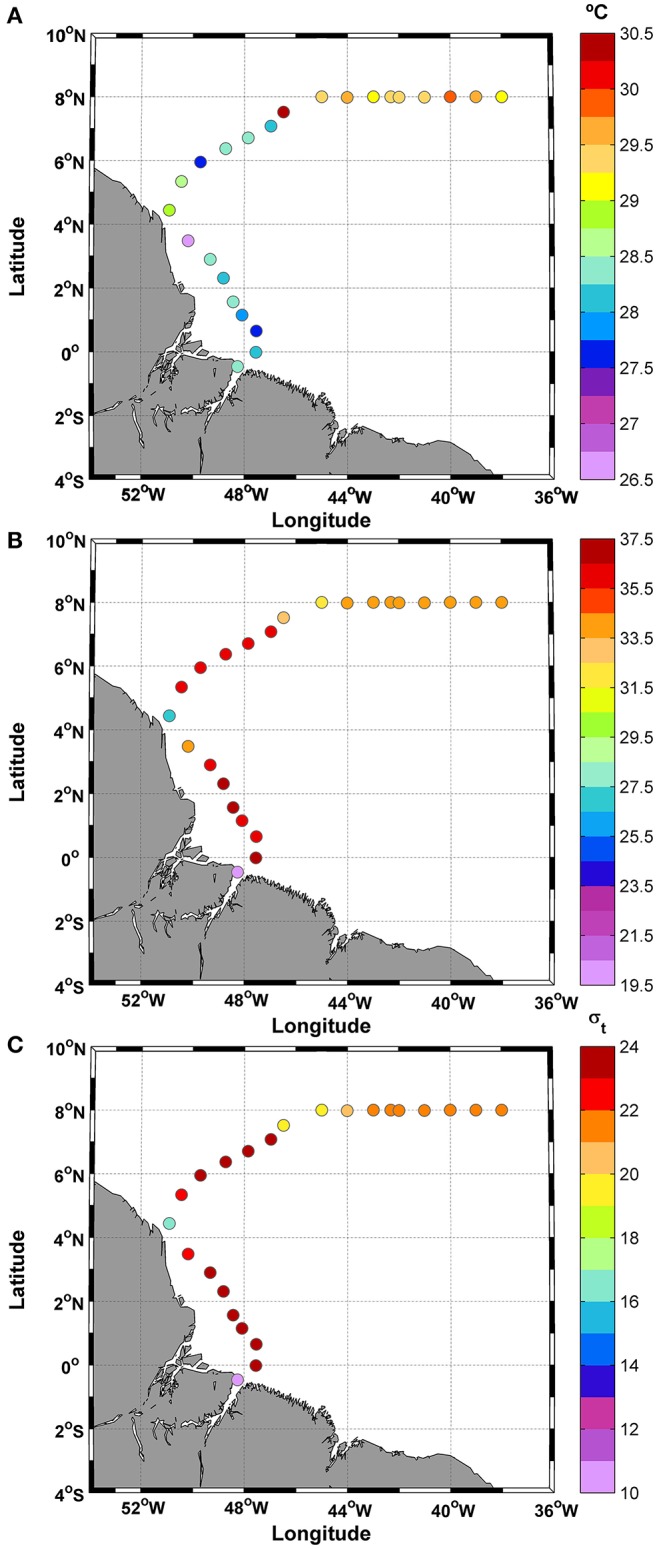
Distribution of **(A)** SST; **(B)** SSS; and **(C)** σ-t along the ship track during the CF3 cruise, October 2012.

Overall, a mean SST of 28.7 ± 0.9°C was measured along the cruise track between 50.9° and 38°W (Figure 2A). The SST values varied during the cruise, reaching a maximum difference of 3.9°C and exhibiting a positive linear trend of 0.087°C (*y* = 0.087(SST) + 27.62) along the cruise track. However, no significant differences were found between day and night (*t*-test; *p*: 0.65; α: 0.05). The large temperature range was caused by two extreme values at stations 8 (26.59°C) and 15 (30.48°C) (Figure 2A). The removal of these SST values from the observations does not significantly alter the overall mean value (average without extreme values: 28.7 ± 0.7°C). In addition, the coefficient of variation (CV) was 0.03%.

The SSS showed typical brackish water values during most of the cruise (>50% of the samples), as reflected in the mean value of only 34.1 ± 3.7 practical salinity units (psu). The day/night cycle did not affect the salinity values (*t*-test; *p*: 0.24; α: 0.05). The salinity data showed a higher CV than temperature (0.11%) (Figure 2B).

The density values (σ-t) (average: 21.5 ± 2.8) were low along the track. For example, the SST average for the track (28.7°C) and SSS = 35 correspond to an σ-t of 22.16, whereas the highest SST value (30.5°C) and SSS = 35 correspond to an σ-t of 21.56. The observations during this period showed low density values typical of brackish waters in the initial and final parts of the transect (Figure 2C). The track showed a minimum σ-t value of 10.86 at station number 1. This station is located near the coast at 0.46°S and 48.25°W (Figure 1).

### Nutrients

**Figure 3:**
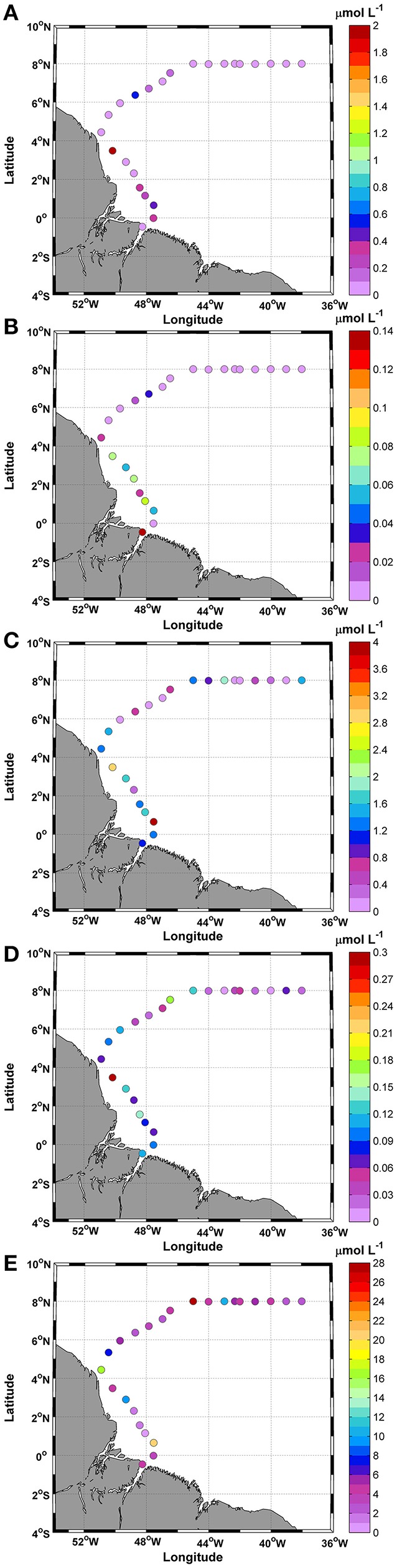
Distributions of **(A)** Ammonia; **(B)** Nitrite; **(C)** Nitrate; **(D)** Phosphate; and **(E)** Silicate along the ship track during the CF3 cruise, October 2012.

The nutrient analysis indicated low ammonia (<2.0 μmol L^−1^) and nitrite (<0.15 μmol L^−1^) values. The highest values were measured at station 8, together with the lowest temperature (Figures 2A, 3A,B). Ammonia and nitrite did not show significant differences between day and night (*t*-test; *p*: 0.41; α: 0.05 and *t*-test; *p*: 0.13; α: 0.05, respectively).

The nitrate concentrations were higher than the ammonia and nitrite concentrations during the entire track. The highest concentrations were in stations 3 and 8 (3.85 and 2.90 μmol L^−1^, respectively). The average concentration was 1.02 ± 0.9 μmol L^−1^ with a CV of 0.94%, and no significant differences were observed between day and night (*t*-test; *p*: 0.48; α: 0.05) (Figure 3C).

The sum of the concentrations of dissolved inorganic nitrogen compounds (ammonia + nitrite + nitrate = DIN) was calculated to obtain a relationship with phosphate concentrations.

The DIN average obtained for this period was 1.25 ± 1.2 μmol L^−1^. The contributions of these compounds to DIN showed that the nitrate represented the greatest contribution, at 82.2%, followed by ammonia at 15.7% and nitrite at 2.1%.

The phosphate (${\text{PO}}_{4}^{-}$) concentrations featured an average of 0.1 ± 0.06 μmol L^−1^ for the study period (Figure 3D). No significant differences were observed between day and night (*t*-test; *p*: 0.52; α: 0.05). The highest value (0.3 μmol L^−1^) was recorded at station 8 of the transect. The average ratio between DIN and ${\text{PO}}_{4}^{-}$ was 15:1. Large variations in this ratio were observed during the study, mainly due to the low values of some compounds. The calculated CV was 1.6%, and the standard deviation was 40.4. The silicate (${\text{SiO}}_{2}^{-}$) showed three considerable peaks along the cruise track (Figure 3E). The mean concentration obtained was 6.4 ± 6.4 μmol L^−1^, and the maximum observed value was 27.0 μmol L^−1^ at station 16. No significant differences were observed between day and night (*t*-test; *p*: 0.68; α: 0.05).

The mean ratio of ${\text{SiO}}_{2}^{-}$:${\text{PO}}_{4}^{-}$ was 80.5:1, while the mean ratio of DIN:${\text{SiO}}_{2}^{-}$ was 0.2:1. Thus, the DIN:${\text{SiO}}_{2}^{-}$:${\text{PO}}_{4}^{-}$ ratio in this study (15:80.5:1) deviated from the Redfield 16:15:1 (DIN:${\text{SiO}}_{2}^{-}$:${\text{PO}}_{4}^{-}$) relationship for ocean waters.

### DO, saturation rate and AOU

**Figure 4:**
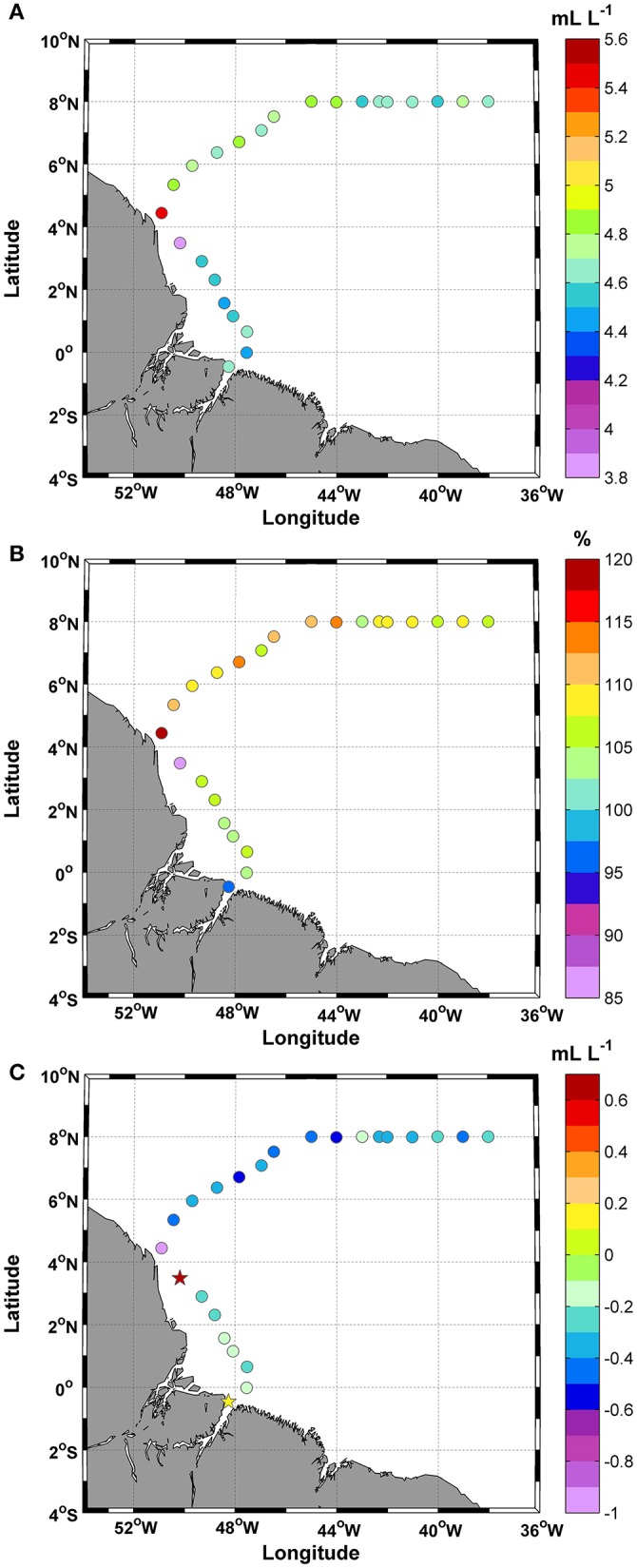
Distributions of **(A)** Dissolved oxygen (DO); **(B)** Saturation of DO (%); and **(C)** Apparent oxygen utilization (AOU) along the ship track during the CF3 cruise, October 2012.

The DO concentrations were always >3.8 mL L^−1^, and the mean value was 4.7 ± 0.2 mL L^−1^ (Figure 4A). The CV was 0.06% for this study. The highest concentration was observed at station 9 (5.5 mL L^−1^). During the day/night cycle, no significant differences were observed (*t*-test; *p*: 0.41; α: 0.05). Based on the percent saturation of DO results, 91% of the samples exhibited supersaturation values (>100%), whereas only two samples (stations 1 and 8) exhibited undersaturation values (Figure 4B). The mean value of the percent saturation was 107.0 ± 6.4%. Similar to the percent saturation, the AOU values at stations 1 and 8 differed from those of other stations. Positive values were observed at stations 1 and 8 (+0.2 and +0.7 mL L^−1^, respectively), while the remaining 91% of the samples showed negative values. The mean value was −0.3 ± 0.2 mL L^−1^, and the lowest values were observed at stations 9–17 (middle section of the track) (Figure 4C).

### The CO_2_ system

**Figure 5:**
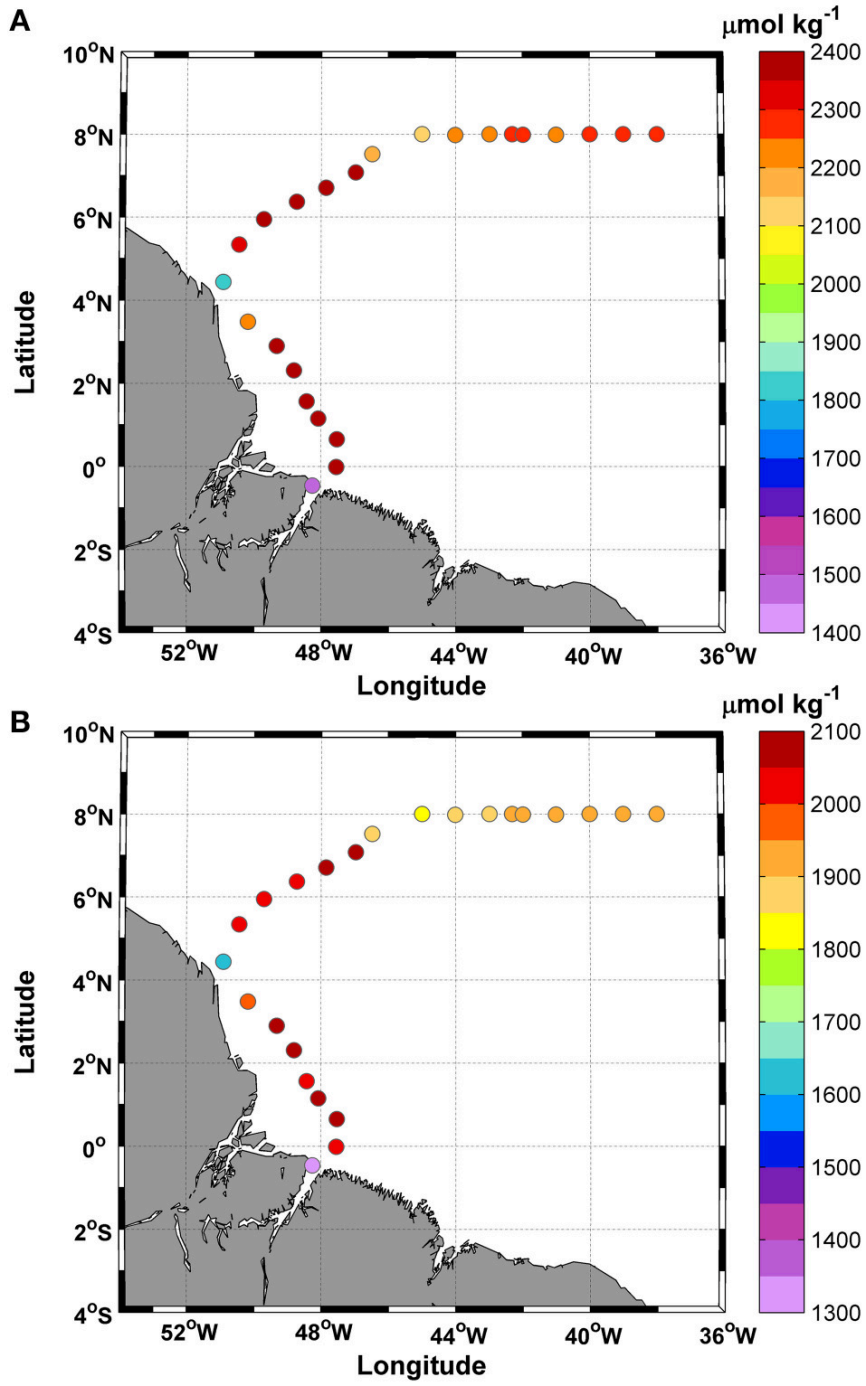
Distributions of **(A)** Total alkalinity (TA); and **(B)** Dissolved inorganic carbon (DIC), along the ship track during the CF3 cruise, October 2012.

Total alkalinity (TA) concentrations varied between 1,450 and 2,394 μmol kg^−1^, with the lowest values occurring at stations 1 and 9 (Figure 5A). The mean TA value was 2,248 ± 212 μmol kg^−1^. TA showed a conservative gradient with salinity from the lowest SSS value observed at station 1 (19.7 psu) to typical oceanic values (max: 36.6 psu). The linear regression obtained for the data was TA = 56.5 ± 0.8 (SSS) + 322.5 ± 28.1, with an *r*^2^ = 0.99. The error in the predicted TA is 14.7 μmol kg^−1^.

DIC concentrations followed the same spatial pattern as TA (Figure 5B) and ranged between 1,303 and 2,062 μmol.kg^−1^. The mean DIC value was 1,935 ± 169 μmol kg^−1^. DIC and SSS were also highly correlated (DIC = 44.7±1.2 (SSS) + 409.4 ± 40), with an *r*^2^ = 0.98. The prediction error was 20.8 μmol kg^−1^.

High values (>2,000 μmol kg^−1^) were observed in transects 2–8 and 10–14 and were associated with a specific range of SSTs (27.6–28.6°C).

**Figure 6:**
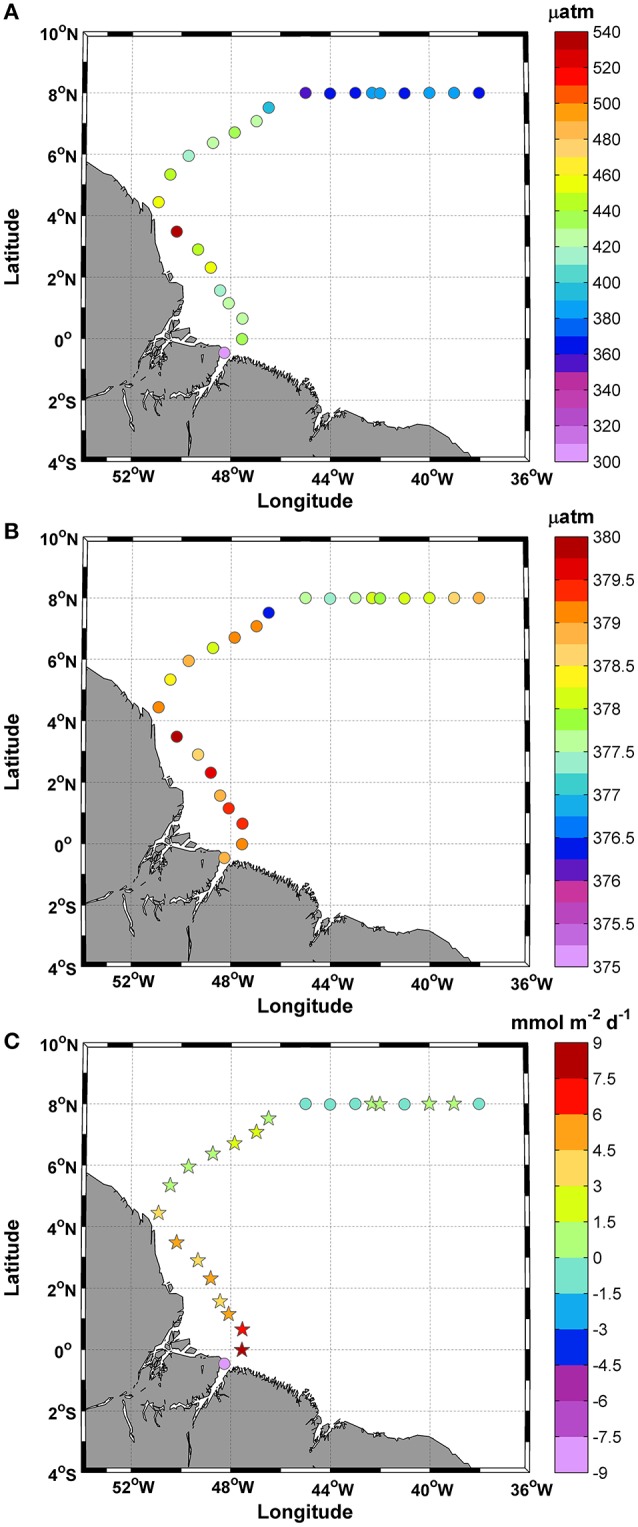
Distributions of **(A)** fCO_2_sw; **(B)** fCO_2_atm; and **(C)** Calculated CO_2_ fluxes along the ship track during the CF3 cruise, October 2012. The circle and star symbols in 6C indicate negative and positive values, respectively.

The fCO_2_sw values calculated from the measured DIC and TA values varied between 304.1 and 543.5 μatm during the study period, with a mean value of 407.8 ± 47.8 μatm (Figure 6A). In October 2012, the average atmospheric fCO_2_ was 378.5±0.8 μatm. Regions of low saturation were observed at station 1 and where the SSS values were <35 in the final portion of the track (Figure 6B).

The fCO_2_sw values did not show a strong correlation with SSS (*r*^2^ = 0.2), especially for SSS values <35. We observed three stations (1, 8, and 9) with values that plot off the trend line. Without these values, the correlation between fCO_2_ and SSS rises to *r*^2^ = 0.8.

The calculated CO_2_ fluxes varied between −8.6 and +8.4 mmol m^−2^ d^−1^, while the mean was +1.6 ± 3.4 mmol m^−2^ d^−1^, and the CV = 2.1% (Figure 6C). The variations in the CO_2_ fluxes recorded for this period showed that 75% of the samples acted as CO_2_ sources to the atmosphere and that 25% acted as CO_2_ sinks. Most positive CO_2_ fluxes were associated with temperatures in the range of 27.6–28.6°C.

### Plankton communities and Chl-*a*

**Figure 7:**
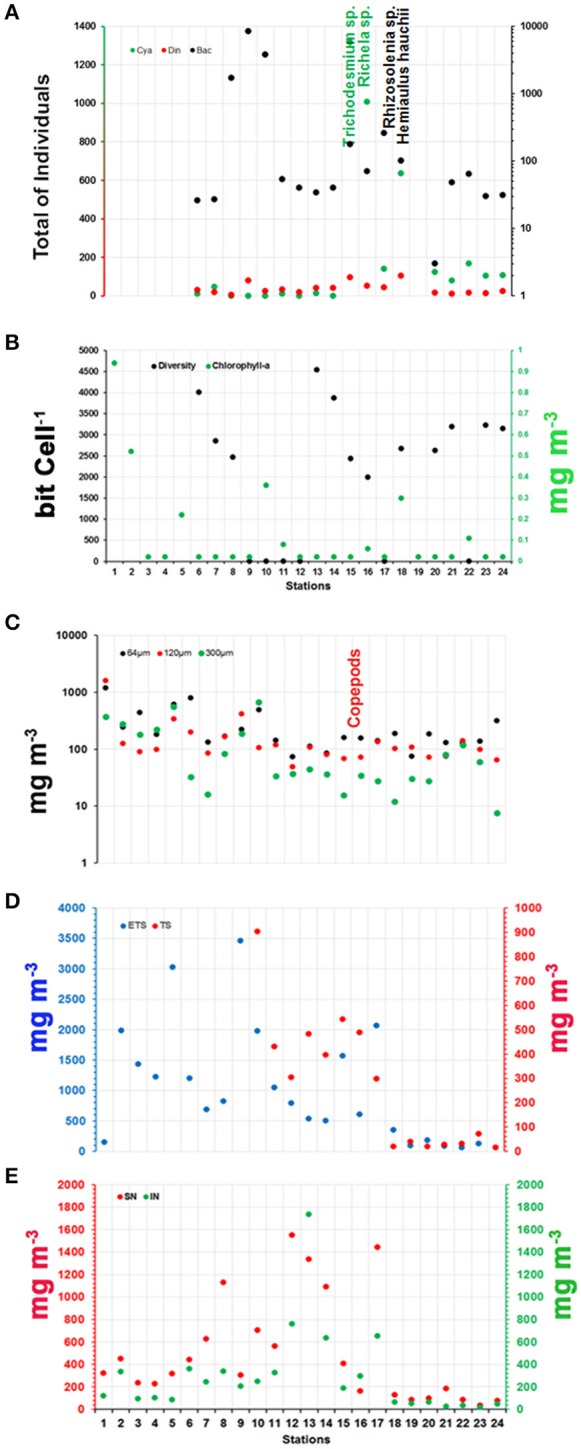
Distributions of **(A)** Phytoplankton groups; **(B)** Diversity and Chl-a; **(C)** Zooplankton groups; **(D)** Vertical biomass of zooplankton; and **(E)** Neuston biomass along the ship track during the CF3 cruise, October 2012. The most representative species of each group (phytoplankton and zooplankton) are shown in vertical orientation in **(A,C)**, respectively. The location of the species in the figure indicates the region of most representative of them.

Phytoplankton communities displayed a strong gradient in terms of total individuals. The highest values were observed at stations 9 and 10 (>3,800 individuals), and relatively high values were also observed at stations 8, 15, and 16 (1,000 < individuals < 3,800). The mean value for total phytoplankton was 1,082 ± 2,100 individuals (Figure 7A).

The phytoplankton community in the studied area consisted of 145 different species in three phyla: Miozoa (57%), Bacillariophyta (37%) and Cyanobacteria (6%). The phyla Miozoa and Bacillariophyta represented the 94% of the planktonic flora diversity. Dinoflagellates and diatoms were present at all stations, whereas Cyanobacteria exhibited relatively high abundances at stations 15–24 (Figure 7A). Diatoms were most abundant, presenting 15,053 individuals during the period studied, whereas the Cyanobacteria were the second most abundant with 3,760 individuals. Dinoflagellates were present at all stations but at lower abundances than the other phyla (666 individuals) (Figure 7A).

The spatial diversity had an average value of 2,059 ± 1,600 bits cell^−1^ and ranged between 2.53 and 4,534 bits cell^−1^. The greatest diversity was at stations 6 and 13 (4,009 and 4,534 bits cell^−1^, respectively), while the lowest diversity was at stations 9, 10 and 22 (2.67, 2.56, and 2.53 bits cell^−1^, respectively) (Figure 7B). Cyanobacteria vs. dinoflagellates showed a slight correlation (Pearson correlation; ρ: 0.58), whereas diatoms showed significant negative correlations with σ-t (ρ: −0.80), DIC (ρ: −0.84), TA (ρ: −0.78) and SSS (ρ: −0.83) and a significant positive correlation with Chl-*a* (ρ: 0.55). Chl-*a* concentrations varied from 0.02 to 0.94 mg m^−3^, with a mean concentration of 0.12 ± 0.2 mg m^−3^ and a CV of 1.8%. Low values were recorded for most of the track (16 samples with 0.02 mg m^−3^; 66%). The highest concentrations were observed in stations 1 and 2 (0.94 and 0.52 mg m^−3^, respectively) (Figure 7B). During the day/night cycle, no significant differences were observed (*t*-test; *p*: 0.20; α: 0.05). Chl-*a* concentrations also showed a negative correlation with TA, DIC, SSS, and σ -t (Pearson correlation; ρ: −0.57; −0.57; −0.57, and −0.55, respectively).

The zooplankton was composed of 10 phyla: Protozoa, Cnidaria, Mollusca, Annelida, Crustacea, Bryozoa, Brachiopoda, Chaetognatha, Echinodermata, and Chordata. A total of 178 taxa were identified based on the lowest distinguishable taxonomic ranking. Holoplankton dominated in the study area, representing nearly 85% of the zooplanktonic biomass (It means 85% of total identified organisms–biodiversity). The most diverse and abundant group was Copepoda, with 130 species and accounting for more than 60% of the zooplankton. Two zooplankton communities were identified in the area: a low-diversity, high-abundance coastal community present at inshore stations and a high-diversity, low-abundance oceanic community at offshore stations. A maximum abundance zone occurred around the shelf break. Copepoda played a central role in the marine food web of the area. The Copepoda species that dominated offshore areas were *Undinula vulgaris, Euchaeta marina, Nannocalanus minor, Clausocalanus furcatus, Scolecithris danae, Calocalanus pavo, Corycaeus (Corycaeus) speciosus, Farranula gracilis*, and *Oithona plumifera*. The Copepod species that were abundant in neritic waters and indicative of the NBC were *Subeucalanus pileatus, Rhincalanus cornutus*, and *Temora stylifera*. These species of copepods are known by the literature (Boltovskoy, 1981, 1999) to occur is this water mass. Meroplankton, mainly zoea-stage larvae of brachyuran crabs, were abundant at coastal stations under stronger plume influence.

Zooplankton biomass (64, 120, and 300 μm mesh nets) showed a huge range, from 7.6 to 1,605 mg m^−3^. Figure 7C shows the spatial distribution of the three compartment types (net size). The lowest values of biomass were associated with the 300 μm mesh (average: 130 ± 174 mg m^−3^; green circles in Figure 7C), while the highest values were associated with the 64 μm mesh (average: 270 ± 265 mg m^−3^; black circles in Figure 7C). The biomass values showed significant differences among the nets (64, 130, and 300 μm, Kruskal-Wallis ANOVA; *p*: 0.0001), mainly between the 64 and 300 μm nets (Dunn test; *p*: 0.0001).

Vertical hauls were conducted with plankton nets with a 200 μm mesh size to sample two depth strata (deep hauls and shallow hauls through the upper mixed layer). The vertical tows with the 200 μm mesh net showed high biomass values (>1,000 mg m^−3^) for the upper mixed layer, while the deep hauls yielded much lower values, always less than 1,000 mg m^−3^ (Figure 7D). The mean values were 1,003 ± 946 and 271 ± 269 mg m^−3^ for shallow and deep hauls, respectively. The highest deep haul values were observed between stations 10 and 17. Samples from shallow and deep hauls showed significant differences (*t*-test; *p*: 0.0006) for the study period.

Neuston tows were also conducted at all stations. Two superimposed nets were used, one at the air-water interface (upper neuston) and the other sampling from 7.5 to 22.5 cm below the interface (lower neuston). The highest biomass value was observed at station 13 for the lower net (1,734 ± 371 mg m^−3^; Figure 7E). However, the highest biomass value in the upper net (average: 500 ± 467 mg m^−3^) was associated with a lower net biomass value of 293 ± 371 mg m^−3^. The highest combined values for the upper and lower nets (>1,000 mg m^−3^) were observed at stations 8, 12, 13, 14, and 17 (Figure 7E). The two sets of samples (lower and upper nets) did not show significant differences (*t*-test; *p*: 0.09) for the studied period.

### Cluster analysis and PCA

**Figure 8:**
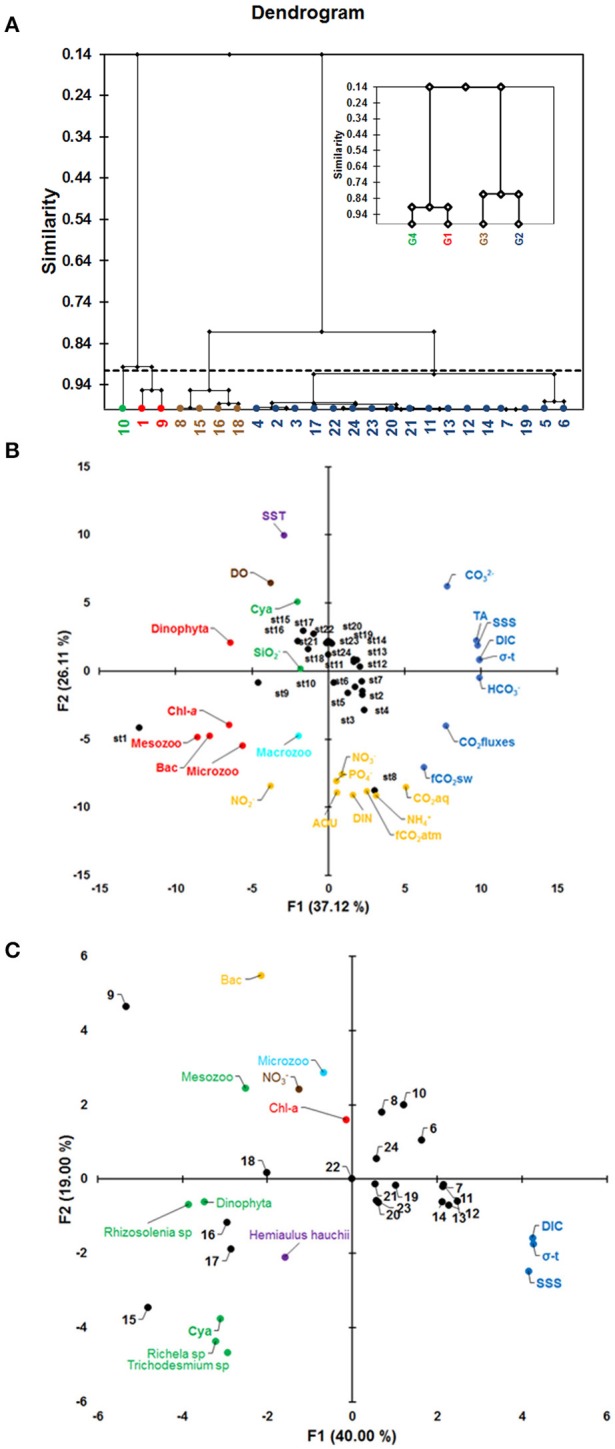
**(A)** Clustering diagram (G1 = group 1, in red color; G2 = group 2, in blue color; G3 = group 3, in brown color and G4 = group 4, in green color); **(B)** Principal component analysis (PCA) with all variables found along the full ship track during the CF3 cruise, October 2012; and **(C)** PCA with biological groups in the offshore NECC region. The acronyms correspond to those specified in Table 1 for PCA analysis.

The cluster dendrogram shown in Figure 8A represents the results of the algorithm forming groups of observations and then subgroups of observations. The algorithm successfully grouped all the observations. The dotted line represents the automatic truncation, leading to four groups (Figure 8A). Groups 1 and 4 (displayed in red and green, respectively) are less homogeneous than the others. Group 2 (displayed in blue) is more homogeneous than groups 1 and 4 (minor variance). Stations 1, 9 and 10 in groups 1 and 4 (red and green colors) were associated with high values of phytoplanktonic and zooplanktonic biomass, low values of density (σ-t), low values of DIC and high values of ${\text{NO}}_{3}^{-}$, whereas group 2 showed the opposite characteristics of groups 1 and 4. Group 2 features the largest number of stations (17). Group 3 contains 4 stations, whereas group 4 features only one station (station 10).

The PCA identified two leading modes that account for 63% of the variability. The first mode (37%) opposes HC${\text{NO}}_{3}^{-}$, DIC, σ-t, SSS, TA, C${\text{CO}}_{3}^{2-}$, CO_2_ fluxes and fCO_2_sw to Mesozooplankton (Mesozoo), Bacillariophyta (Bac), Chl-*a* (Chl-*a*), Dinophyta and Microzooplankton (Microzoo), as shown in the bi-plot of the first two factors (Figure 8B). The principal components (PC) are plotted as a function of the twenty-four stations measured in October 2012 (the green, brown and cyan colors correspond to the third, fourth and fifth components, respectively, in Figures 8B and Table 1). The factor loadings of the five modes are given in Table 1. The first principal component (PC1) highlights the cross-shelf variability, with strong differences between stations 1 and 9 and the other stations (blue and red in Figure 8B). The second mode explains 26% of the variance and is dominated by nutrients (${\text{NH}}_{4}^{+}$, ${\text{SiO}}_{2}^{-}$, ${\text{NO}}_{3}^{-}$, DIN, and ${\text{PO}}_{4}^{-}$), AOU, CO_2_aq and fCO_2_atm (orange in Figure 8B) opposed to SST (+ axis) (Figure 8B). PC3 (10%) is characterized by ${\text{SiO}}_{2}^{-}$ and Cyanobacteria (Cya) (green in Figure 8B). PC4 (7.8%) is characterized by DO, which does not appear to be correlated with any other parameter on this axis (brown in Figure 8B). Macrozooplankton appears in the fifth mode and shows no correlation with any other parameters of this analysis (cyan in Figure 8B).

**Table 1:** Loading factors of the 5 principal components (PCA analysis).

**Parameters**	**F1**	**F2**	**F3**	**F4**	**F5**
**SSS**	**0.97**	0.16	−0.08	−0.04	0.15
**TA**	**0.96**	0.19	−0.11	−0.04	0.15
**DIC**	**0.98**	0.07	−0.09	0.02	0.11
**fCO**_2_**sw**	**0.62**	−0.58	0.13	0.35	−0.21
σ**-t**	**0.98**	0.07	−0.10	−0.02	0.14
**CO**_2_**fluxes**	**0.76**	−0.33	0.07	0.32	0.15
**${\text{HCO}}_{3}^{-}$**	**0.98**	−0.04	−0.07	0.07	0.07
**${\text{CO}}_{3}^{2-}$**	**0.77**	0.52	−0.16	−0.20	0.25
**Chl-a**	−**0.64**	−0.33	−0.30	−0.21	0.40
**Microzoo**	−**0.56**	−0.45	−0.37	−0.06	0.36
**Mesozoo**	−**0.85**	−0.40	−0.28	−0.06	0.02
**Dinophyta**	−**0.64**	0.18	0.37	0.07	0.04
**Bac**	−**0.77**	−0.39	−0.03	0.44	−0.05
**SST**	−0.29	**0.83**	0.17	−0.12	0.05
**${\text{NH}}_{4}^{+}$**	0.31	−**0.76**	0.32	−0.19	−0.26
**${\text{NO}}_{2}^{-}$**	−0.38	−**0.70**	−0.22	−0.05	−0.11
**${\text{NO}}_{3}^{-}$**	0.08	−**0.63**	0.50	−0.01	0.46
**DIN**	0.16	−**0.75**	0.49	−0.07	0.27
**${\text{PO}}_{4}^{-}$**	0.05	−**0.67**	0.43	−0.18	−0.13
**AOU**	0.05	−**0.74**	−0.05	−0.65	−0.08
**fCO**_2_**atm**	0.25	−**0.73**	−0.39	0.29	−0.09
**CO**_2_**aq**	0.50	−**0.70**	0.13	0.35	−0.24
**Cya**	−0.20	0.43	**0.71**	−0.31	0.08
**${\text{SiO}}_{2}^{-}$**	−0.18	0.02	**0.68**	0.36	0.31
**DO**	−0.38	0.54	0.05	**0.74**	0.02
**Macrozoo**	−0.20	−0.39	−0.30	0.19	0.63

*The shading corresponds to factor 1 (positive-blue and negative-red). The following values correspond to factor 2 (positive-purple and negative-yellow), factor 3 (positive-green), factor 4 (positive-brown) and factor 5 (positive-cyan). The values in bold indicate a significant correlation*.

## Discussion

The results obtained from this cruise improved our understanding of the processes and organisms responsible for carbon and nutrient cycling along this large-scale river-ocean continuum. The contributions of the different factors explaining the biogeochemical pathways and carbon fluxes in the oceanic area under the influence of the Amazon River plume are extremely complex. Thus, we focused our discussion on systematically linking the variability in the physical and biological processes to the biogeochemistry and carbonate system in this huge area.

### Surface circulation and distribution of SST and SSS

The hydrographic results revealed very different conditions in the study area, ranging from shallow coastal conditions to offshore areas. The distribution of the sampling stations up to 8°N-38°W (station 24) showed that a large number of these stations were located within the complex system of currents in the region, but most were affected by two main currents, the NBC and the NECC. The NECC intensifies during summer (June, July, and August) and fall (September, October, and November), while the NBC retroflects in this period. The NBC retroflection (Wilson et al., 2002), a result of the vorticity balance, is shown in Figure 1B as the clockwise gyre centered at 5°N, 45°W. The retroflection is weaker in spring (March, April, and May) and stronger in fall (September, October, and November) (Urbano et al., 2008; Lefevre et al., 2014). We observed the retroflection in fall (October) (Figure 1B). This zonal current system plays an important role in modulating the heat flux in the tropical Atlantic. During this period, SSTs varied considerably along the transect, but this variation did not influence the overall mean value of 28.7°C. Warmer waters (>28°C) from the Amazon and the Pará rivers were widely observed along the inner continental shelf, the Amazon and Pará river mouths and offshore areas (Figure 1C). The values observed here are similar to the historic SSTs in the region 0°–9°N, 36°–52°W for the period 1958–2011 (28.0 ± 0.3°C) (Yu et al., 2008) and are in accordance with the findings of other authors, such as Lefevre et al. (2014) and Urbano et al. (2008).

The SST values showed an inverse relationship with nutrients, AOU, and dissolved CO_2_ (CO_2_aq) (Figure 8B) but were not correlated with SSS values, as indicated by Ibánhez et al. (2015) for the plume of the Amazon River. The differences between SST and SSS can be explained by the strong amplitude of SSS (higher than 8 psu) whereas, SSTs exhibited differences of less than 2°C. Thus, over half of the 24 samples of the CF3 cruise (54%) used here showed a minimum SSS lower than 35 psu (Figure 2B).

According to Ibánhez et al. (2016), a high correlation exists between rainfall and SSS in the plume region, and this relationship can affect >16% of the Amazon River plume area. The spatial extent of the influence of brackish waters was highly variable. Low SSS values were found at 8°N, 38°W, indicating the influence of the local surface water circulation on the spread of Amazon River waters in the WTNA (Figures 1F, 2B). The rainfall in October 2012 in the vicinity of the CF3 transect was approximately 0.5 mm h^−1^ (Figure 1E).

### SSS and carbonate system parameters

The SSS was also highly positively correlated with the carbonate system parameters (HC${\text{NO}}_{3}^{-}$, DIC, TA, C${\text{CO}}_{3}^{2-}$, CO_2_ fluxes and fCO_2_sw). According to Koffi et al. (2010); Lefèvre et al. (2010), and Bonou et al. (2016) in the WTNA, SSS has been shown to be highly correlated with TA and DIC.

Both relationships (TA-SSS and DIC-SSS) are quite robust, as shown by the good agreement between the regression and the available observations from previous cruises (TA = 56.46 × SSS + 322.48; *r*^2^ = 0.99 and DIC = 44.74 × SSS + 409.42; *r*^2^ = 0.98). The TA–SSS and DIC–SSS regressions were performed for salinities greater than 19. Our TA-SSS data are in agreement with the relationship of Lefèvre et al. (2010).

Ternon et al. (2000) reported TA–SSS and DIC–SSS slopes of 58.85 and 49.48 μmol kg^−1^, respectively, using cruise data at the Amazon River mouth. These TA–SSS and DIC–SSS slopes are very close to those obtained in our study (56.4 and 44.7 μmol kg^−1^, respectively).

Salinity and fCO_2_sw values lower than those in the literature were observed during the CF3 cruise and were due to the influence of Amazon River water and rainfall. The maximum and minimum fCO_2_ values were associated with SSS values of <35, both of which were observed in areas influenced by the Amazon plume. The fCO_2_sw values within the Amazon River plume are significantly correlated with SSS (Lefèvre et al., 2010; Ibánhez et al., 2016). However, Ibánhez et al. (2015) noted that, brackish waters (SSS<35) transported by the NBC and collected south of 8°N showed the highest SSS-fCO_2_sw discrepancy among consecutive cruises between 2006 and 2013 (the period covering the CF3 cruise). According to these authors, many surface eddies present in this region of the NBC retroflection (a significant path of water mass transport in the area) (Ffield, 2005) may be responsible for the spatial variability found near the coastal zone.

The SSS-HC${\text{NO}}_{3}^{-}$ and SSS-C${\text{CO}}_{3}^{2-}$ correlations were positive (*r* = 0.97 and *r* = 0.88, respectively) and were linked to the concentrations of TA and DIC. They represented 88% and 11.5% of the DIC, respectively, while the remaining 0.5% corresponded to CO_2_aq, which showed a low correlation with SSS. According to Richey et al. (1990) and Cooley and Yager (2006), the Amazon River mainstream DIC load represents 82% of the HC${\text{NO}}_{3}^{-}$ and 18% of the CO_2_aq in the river. CO_2_aq is mainly controlled by pH, SST and SSS and the concentrations decrease along the salt gradient.

The CO_2_ fluxes-SSS correlation was positive (*r* = 0.7) along the ship track. The large variation (17 mmol) was forced by stations 1 and 2, where the magnitude of the CO_2_ fluxes was also influenced by the intensity of the winds, with speeds of 7.3 and 9.0 m s^−1^, respectively. The majority of positive CO_2_ fluxes were associated with temperatures of 27.6–28.6°C and salinities of 34–37 psu. Our study showed that 75% of the 24 stations exhibited CO_2_ oversaturation and that only 25% of the sites exhibited undersaturation. These results may be associated with the route followed by the CF3 cruise. Figure 1F shows that a portion of the stations are located within a region with SSS values of >35 (St. 2–7). Another factor that can affect the direction of the CO_2_ fluxes is light. The amount of light affect the growth of phytoplankton in the oceans, and their rate of photosynthesis increases in proportion to the light intensity. During the CF3 cruise, 33.3% of the samples were obtained at night. During this period, productivity is less than respiration, and heterotrophic processes in the water column can increase the CO_2_aq and decrease the DO concentrations. Beyond the retroflection region, we observed a greater fluctuation around zero (oversaturation/undersaturation) in the CO_2_ fluxes.

According to Ibánhez et al. (2015), CO_2_ undersaturation in the brackish waters transported by the NBC through retroflexion to the north occurs during the second half of the year. In this region and during the boreal autumn, the Amazon discharge and the location of the ITCZ can lead to undersaturation (Lefèvre et al., 2010). The migration of the ITCZ is consistent with the salinity distribution with low salinities located north of 2°N from July to December. The amount of rainfall observed in this study was 10 times lower than that observed by Lefèvre et al. (2010) (Figure 1E).

According to Lefèvre et al. (2010), low salinities (SSS < 35) are encountered from July to December between 2° and 8°N. Similar values were recorded by the CF3 cruise during the month of October. Additionally, the low-salinity region is associated with a decrease in ΔfCO_2_. According to Lefèvre et al. (2010), the eastward propagation of Amazon waters is unlikely to explain the CO_2_ undersaturation observed throughout the year because Amazon waters might reach 25°–30°W only in boreal autumn, depending on the strength of the NECC.

The ΔfCO_2_ values varied strongly during the CF3 cruise, passing from highly undersaturated conditions in the coastal region (1°S) to oversaturated conditions between 0° and 7°N. During the final part of the ship track (45°–38°W), the ΔfCO_2_ varied slightly between positive and negative values (8°N). Within this region, the CO_2_ fluxes varied slightly between source and sink, whereas the fCO_2_sw values were very close to atmospheric fCO_2_ (<5 μatm for ΔfCO_2_).

Along the CF3 ship track in October, the calculated CO_2_ fluxes (+1.6 ± 3.4 mmol m^−2^ d^−1^) had the same magnitude as the values reported by Lefèvre et al. (2010) and Ibánhez et al. (2015) for the same period.

### Nutrients

Riverine nutrients and *in situ* organic matter mineralization support primary production in the offshore plume (DeMaster and Pope, 1996; Subramaniam et al., 2008; Yeung et al., 2012). The effects of freshwater inputs on coastal CO_2_ and the carbonate system dynamics occur via direct inputs of DIC or through enhanced primary production due to river-borne nutrient inputs (Kitidis et al., 2012).

To further examine the effects of nutrient concentrations on the carbonate system of coastal waters, N^*^ and DINxs indices were applied to determine the relative abundances of DIN and ${\text{PO}}_{4}^{-}$ in the study region.

The nitrate concentrations were higher than the ammonia and nitrite concentrations throughout the cruise.

Nitrate represented 82.2% of the DIN, ammonia represented 15.7% of the DIN, and nitrite represented only 2.1% of the DIN. Thus, the average DIN:phosphate ratio was 15:1, although this ratio varied during the study due to the low values of some compounds.

The DIN compounds showed relative abundances lower than the limits of the indices DINxs and N^*^ (average: −0.2 and 2.6, respectively) (Table 2). Negative DINxs values (or N^*^ values < 2.9 μmol L^−1^) indicate a deficit of N relative to P with respect to the requirements for Redfieldian production of organic matter. The values calculated are consistent with those reported by Hansell and Follows (2008) for the tropical Atlantic. Furthermore, the Redfieldian ratios suggest that the Amazon is an important source of “excess” PO_4_− (DIN:PO_4_− < 16) to the WTNA. The compounds are transported thousands of kilometers offshore via the plume. The plume ranges from 5 to 25 m thick (Coles et al., 2013) and supplies allochthonous Si and ${\text{PO}}_{4}^{-}$ to offshore regions in the tropical Atlantic. This input of plume-derived Si and ${\text{PO}}_{4}^{-}$ into the nitrogen-limited open ocean, with Si:DIN and ${\text{PO}}_{4}^{-}$:DIN ratios in excess of those typically needed by phytoplankton, creates a distinct niche for N_2_ fixation by diatom-diazotroph associations (DDAs), leading to enhanced primary production in this region (Subramaniam et al., 2008). A key hypothesis advanced by Subramaniam et al. (2008) is that DDAs represent an effective biological pump in tropical river plumes. In coastal regions, DIN derived from inland waters is rapidly consumed, leaving an extensive area (~10^6^ km^2^) with lower salinity and excess dissolved ${\text{SiO}}_{2}^{-}$ and ${\text{PO}}_{4}^{-}$ concentrations relative to Redfield-Brzezinski stoichiometry (i.e., C:Si:N:P = 106:15:16:1) in the tropical Atlantic (Brzezinski, 1985; Shipe et al., 2007; Subramaniam et al., 2008). In the study region, the ratio ${\text{SiO}}_{2}^{-}$:DIN:${\text{PO}}_{4}^{-}$ showed a Redfield-Brzezinsky stoichiometry of 15:80.5:1. The Redfield stoichiometry was lower than 16, indicating that nitrogen was the limiting factor in this region. Plots of nutrients vs. salinity are shown in Figures 1SA–C.

**Table 2:** DINxs and N^*^ values along the ship track during the Camadas Finas III (CF3) cruise.

**Station**	**DINxs**	N^*^
1	−0.62	2.28
2	−0.04	2.86
3	2.73	5.63
4	0.41	3.31
5	−1.03	1.87
6	−0.95	1.95
7	−0.34	2.56
8	−1.91	0.99
9	0.04	2.94
10	0.10	3.00
11	−1.71	1.19
12	0.08	2.98
13	−0.28	2.62
14	−0.75	2.15
15	−1.92	0.98
16	−0.80	2.10
17	0.42	3.32
18	1.81	4.71
19	−0.57	2.33
20	−0.65	2.25
21	0.13	3.03
22	0.02	2.92
23	−1.09	1.81
24	1.08	3.98

These diagrams show the removal of the ${\text{NO}}_{3}^{-}$, ${\text{PO}}_{4}^{-}$ and ${\text{SiO}}_{2}^{-}$ compounds.

According to removal equation of Noriega et al. (2013) (diagrams in the Supplementary Material), the DIN and ${\text{SiO}}_{2}^{-}$ were removed in most of the areas with brackish waters, especially in areas with salinities of <35 psu, where ${\text{PO}}_{4}^{-}$ was less removed. ${\text{SiO}}_{2}^{-}$ showed a distribution similar to results by Edmond et al. (1981) and Ternon et al. (2000).

#### Relations between ${\text{SiO}}_{2}^{-}$, DIC, and TA concentrations

We used the conservative theoretical mixing lines and the mixing line of observed data for the ${\text{SiO}}_{2}^{-}$, TA and DIC concentrations to obtain predictions of the effects of biological activity in the study region.

Based on a comparison between the regression line fitted to observed data and the conservative mixing line, the maximum depression in ${\text{SiO}}_{2}^{-}$ (Figure 1SC; Supplementary Material) due to biological activity was approximately 43 μmol kg^−1^ (19–62 μmol kg^−1^, respectively), which is in agreement with the highest ${\text{SiO}}_{2}^{-}$ depletion (greater than 30 μmol kg^−1^) reported by DeMaster and Pope (1996) on the Amazon shelf.

To compare the ${\text{SiO}}_{2}^{-}$ and inorganic carbon depletions involved in biological uptake (maximum depressions of 43 and 82 μmol kg^−1^ for ${\text{SiO}}_{2}^{-}$ and DIC, respectively), we need to correct the inorganic carbon depletion for calcification (Figures 1SC, 2S; Supplementary Material). This correction is always DIC-TA/2, according to Zeebe and Wolf-Gladrow (2001).

The observed TA deviation away from the conservative line (71 μmol kg^−1^ for SSS = 19.7, Figure 2S, Supplementary Material) is typical of calcium carbonate (CaCO_3_) production is seawater (Broecker and Peng, 1982). As the change in TA due to carbonate mineral production is twice as large as the change in DIC (Skirrow, 1975) and by neglecting the small changes in TA due to the production and decay of organic matter (Brewer and Goldman, 1976), the organic carbon production corrected for calcification is 82-71/2 = 46 μmol kg^−1^. Thus, ${\text{SiO}}_{2}^{-}$ and inorganic uptake exhibit a molar ratio of ${\text{SiO}}_{2}^{-}$/DIC = 0.9, which is beyond the range (0.15–0.4) of molar silicon/carbon production ratios reported by DeMaster et al. (1996) and Ternon et al. (2000) in waters of the Amazon shelf. This high value (0.9), in comparison to the mean value of 0.13 for low-latitude diatoms (Brzezinski, 1985), highlights the dominant role of diatoms (mainly *Fragillaria sp*. and *Pseudo-nitzschia pungens*) in the primary production in this zone (stations 8, 9, and 10; Figure 1SC and Figure 7A).

### DO and AOU

The decomposition of organic matter changes the concentrations of carbon, nitrogen, phosphorus, oxygen and TA in the ratio 106:16:1:138:–17. We observed areas of DO-supersaturated surface water, indicative of low consumption. DO concentrations were always >3.8 mL L^−1^, and the mean value was 4.7 ± 0.2 mL L^−1^. AOU concentrations along the CF3 ship track ranged between −0.9 and +0.7 mL L^−1^. However, 91% of the samples showed negative values. The mean value was −0.3 ± 0.2 mL L^−1^, and the lowest values were observed at stations 9–17 (middle section of the track). Thus, the sampling period was characterized by negative AOU values (production > respiration). The oxygen supersaturation in the river plume (negative AOU) is evidence of high photosynthetic activity. Other authors (DeMaster et al., 1996; DeMaster and Aller, 2001; Garcia et al., 2006) also reported negative AOU values.

In addition, biological consumption is one of the processes affecting the variability in the carbon parameters in tropical areas (Cooley et al., 2007; da Cunha and Buitenhuis, 2013; Araujo et al., 2014). We separate the stations with SSS<35 and SSS≥35 along the CF3 ship track. A negative average AOU value of −0.3 mL L^−1^ was found for both divisions of salinity. According to the PCA (Section Cluster Analysis and PCA above), AOU and CO_2_aq showed a positive correlation in factor 2 (orange color in Figure 8B). Thus, negative AOU values are associated with higher CO_2_aq concentrations. According to Zeebe and Wolf-Gladrow (2001), the release of CO_2_ to the atmosphere decreases the DIC concentration, while the TA concentration remains constant. This process leads to a rise (drop) in dissolved CO_2_(CO_2_aq), with the opposite change in pH. The AOU values were characteristic of productive regions and indicated that production was greater than respiration at 91% of the CF3 cruise stations.

### Plankton community and Chl-*a*

The phylum Miozoa and Bacillariophyta characterized 94% of the floristic diversity in the planktonic flora. Dinoflagellates and diatoms were present at all stations of the ship track, whereas Cyanobacteria were more abundant at stations 15–24 (Figure 7A). A dominance of diatoms was observed in the region from the river mouth to the beginning of the area affected by the NBC retroflection (Chl-*a* concentrations ranging from 0.02 to 0.9 mg m^−3^). After retroflection, the NECC region is fully oligotrophic, and the most representative groups are Cyanobacteria and Bacillariophyta.

A comparison of Cyanobacteria vs. dinoflagellates showed a slight correlation (Pearson correlation; ρ: 0.58), whereas diatoms showed significant negative correlations with σ-t (ρ: −0.80), DIC (ρ: −0.84), TA (ρ: −0.78), and SSS (ρ: −0.83) and a significant positive correlation with Chl-*a* (ρ: 0.55). Chl-*a* concentrations also showed negative correlations with TA, DIC, SSS, and σ-t (Pearson correlation; ρ: −0.57; −0.57; −0.57, and −0.55, respectively).

Increases in phytoplankton accompanied decreases in σ-t and DIC. Thus, the variations in the phytoplankton community were reflected in the concentrations of the parameters of the carbonate system (DIC and TA). In addition, the stations located within the NBC (stations 6–10) system were associated with the Bacillariophyta group, while stations located within the NECC (stations 15–18) featured greater numbers of Cyanobacteria individuals. However, the diversity of groups was always greater after retroflection (Figures 1B, 7B). Furthermore, the highest productivity values (negative AOU) were associated with these phytoplankton groups.

*Trichodesmium sp*. and *Richelia sp*. were the main species of Cyanobacteria in the region of the NECC. According to Yeung et al. (2012), phytoplanktonic groups varied along the salinity gradient, and specific groups co-varied. For example, the abundance of *Richelia sp*. was associated with that of *Hemiaulus hauckii*. In the NECC region, we observed an association of *Richelia sp*. and *Trichodesmium sp*. with *Hemiaulus hauckii* and *Rhizosolenia sp*. The planktonic cyanobacteria *Trichodesmium sp*. is globally distributed in the tropical and subtropical oceans (Luo et al., 2012), where water temperatures are above 20°C (Detoni et al., 2016). Understanding the global distribution of *Trichodesmium* is particularly important because of its ability to fix molecular nitrogen (N_2_) (Yeung et al., 2012). The diatoms *Hemiaulus hauckii* and *Rhizosolenia spp*. containing the symbiotic *Richelia sp*. (DDAs) represented 21% of the total phytoplankton species at the mesohaline (32 ≤ SSS < 35) stations (15–24). According to Subramaniam et al. (2008), the composition of the phytoplankton community changes along the Amazon River plume from the mouth to the open ocean in response to changing nutrient availability. At low-salinity stations, sufficient ${\text{PO}}_{4}^{-}$, ${\text{SiO}}_{2}^{-}$, and N compounds are available at the surface to support coastal diatom species, and very little N_2_ fixation occurs in these areas (stations 1–9). As the N compounds are assimilated and the plume is mixed with low-nutrient ocean waters, diazotrophs become significant sources of N. The diatom hosts of *Richelia*, the dominant diazotroph at the mesohaline stations, require the ${\text{SiO}}_{2}^{-}$ and ${\text{PO}}_{4}^{-}$ found in the river plume but N is supplied via N_2_ fixation. Farther “downstream,” where river-associated ${\text{SiO}}_{2}^{-}$ and ${\text{PO}}_{4}^{-}$ are depleted, the species composition transitions to that typical of oligotrophic tropical oceans, and the dominant diazotroph is *Trichodesmium*.

Thus, we conclude that the non-conservative changes in DIC discussed above were associated with the Cyanobacteria group and consequently with N_2_ fixation.

The zooplankton was composed of the phyla Protozoa, Cnidaria, Mollusca, Annelida, Crustacea, Bryozoa, Brachiopoda, Chaetognatha, Echinodermata, and Chordata. In total, 178 taxa were identified, considering the lowest taxonomic unit possible for each phylum. This area was dominated by holoplankton, which represented nearly 85%. Copepoda was the most diverse and abundant group with 130 species, accounting for more than 60% of the zooplankton.

The highest biomass was registered at station 5 at a depth of 46 m followed by station 1 at a depth of 9.2 m, which are strongly affected by the river plume. Both stations exhibited blooms of the diatom *Coscinodiscus centralis*. Additionally, station 5 was dominated by a high density of medusa followed by Copepoda *Undinula vulgaris* and *Lucicutia flavicornis* (adults, copepodite and nauplii), and station 1 featured a high density of characteristic estuarine indicator species (*Acartia tonsa, Paracalanus* sp., *Oithona hebes, Euterpina acutifrons*) in addition to numerous Decapoda larvae.

The highest biomass density offshore was registered at station 17 under the plume influence and was dominated by *Clausocalanus furcatus, Oithona plumifera*, and *Oncaea media*. The densities of these species were associated with blooms of *Trichodesmium* sp. in the oceanic area. The neuston biomass densities were higher at the coastal stations 5 and 9 due to medusa blooms and offshore at stations 12, 13, 14, and 17 due to the presence of fish larvae and gelatinous organisms.

Two zooplankton communities were identified in the area: a low-diversity, generally higher-biomass and higher-density coastal community present at inshore stations and a highly diverse, generally low-density oceanic community at offshore stations. A few oceanic stations registered high biomass values due to jellyfish blooms. A maximum biomass/density zone occurs around the shelf break. Throughout the study area, Copepoda play a central role in the marine food web. Meroplankton individuals, mainly Brachyura zoeae, are abundant at coastal stations under plume influence.

According to the multivariate analysis (PCA), zooplankton biomass showed strong correlations with microzooplankton, mesozooplankton, Chl-*a* and Cyanobacteria, whereas macrozooplankton biomass was not correlated with other parameters (Factor 4).

Recently, Conroy (2016) provided direct evidence demonstrating that two DDAs, *Hemiaulus-Richelia* and *Rhizosolenia-Richelia*, are consumed by mesozooplankton. He further showed that calanoid and harpacticoid copepods, as well as some decapod larvae, consume *Trichodesmium*. Additionally, he showed that unicellular cyanobacteria, particularly non-diazotrophic Synechococcus and Prochlorococcus, as well as diazotrophic (unicellular nitrogen-fixing cyanobacteria, or UCYN-A), are consumed by zooplankton, likely as components of aggregates. Grazing on UCYN-A provides an additional and previously undocumented pathway for diazotrophic nitrogen incorporation into the food web.

Thus, we conclude that the changes in DIC and fCO_2_sw and CO_2_ fluxes in the mesohaline stations were also associated with the Cyanobacteria group (phytoplankton) and N_2_ fixation (mesozooplankton). Microzooplankton represent 25% of the total zooplanktonic biomass in this region, while mesozooplankton represent 21%. We have to consider that the biomass of station 1 near the mouth of the river is composed of 18% microplankton and 35.5% mesozooplankton.

### Clustering analysis and PCA

Clustering was included to identify spatial divisions within the CF3 ship track. We included the main parameters of each area: biological (phytoplankton and zooplankton biomass), physical (σ-t) and chemical (${\text{NO}}_{3}^{-}$ and DIC). The differences between the main groups were analyzed according to the similarity within the dendrogram. Groups 1 and 4 (red and green colors in Figure 8A, respectively) are associated with low values of σ-t, high values of phytoplankton biomass (diatoms group) and zooplankton biomass, high ${\text{NO}}_{3}^{-}$ concentrations, and low DIC concentrations. Group 1 and 4 differ in that station 10 has a higher σ-t value than the other 2 stations. Groups 3 and 4 show a smooth similarity differing mainly because of σ-t. These groups show a mix of stations associated with the NBC and NECC region.

The PCA identifies three leading modes that account for 73% of the variability encountered. The first mode (37%) sets HC${\text{NO}}_{3}^{-}$, DIC, σ-t, SSS, TA, C${\text{CO}}_{3}^{2-}$, CO_2_ fluxes and fCO_2_sw (shown in blue in Figure 8B) in opposition to mesozooplankton, Bacillariophyta, Chl-*a*, Dinophyta, and microzooplankton (shown in red), as presented in the bi-plot of the first two factors (Figure 8B). In this analysis, we do not consider species.

We found a significant negative correlation between biological parameters and DIC (Table 1). Other parameters of the carbonate system, such as HC${\text{NO}}_{3}^{-}$, C${\text{CO}}_{3}^{2-}$ fCO_2_, and CO_2_ fluxes, were also negatively correlated with biological groups. On the other hand, we posit that the composition of the phytoplankton community changes along the Amazon River plume from the mouth to the open ocean in response to changing nutrient availability. Nitrogen compounds and phosphates did not show associations in the first mode (shown in orange in Figure 8B). Stations located near the coast (stations 1 and 8) show a strong association with nutrients and AOU (orange in Figure 8B). Diatom groups that increase productivity (negative AOU) and release CO_2_aq dominate this region (orange in Figure 8B). Based on the cluster analysis, the region exhibits segmentation, as a cyanophyte bloom was observed in the NECC region (stations 15–18). We applied a new PCA using the most abundant phytoplankton and zooplankton groups and species in this region. Additionally, we include ${\text{NO}}_{3}^{-}$, DIC, SSS, and Chl-*a*. The results of the multivariate analysis showed a strong association among the cyanophytes *Trichodesmium sp*. and *Richelia sp*., the diatom group *Rhizosolenia* sp. and mesozooplankton (Mesozoo) in the first mode (40%) (shown in green in Figure 8C). These species showed a negative correlation with SSS and DIC (shown in blue in Figure 8C). In addition, ${\text{NO}}_{3}^{-}$, Chl-*a* and microzooplankton (Microzoo) did not show an association with these parameters (Figure 8C). The mesozooplankton is mainly composed of copepods (>60%) in this region.

## Conclusion

The ship track during the oceanographic cruise Camadas Finas III encompassed the outer Amazon River estuary, the alongshore northwestern NBC region, the NBC retroflection area and the eastern NECC plume transport to 38°W. The cruise was purposefully planned to take place during boreal autumn (October 2012), when the dispersal of Amazonian waters forms a brackish plume that can reach 25°W when the NECC is strong.

Hydrographic results showed very different situations, ranging from shallow well-mixed coastal scenarios to offshore areas where low-salinity Amazonian waters induce the formation of barrier layers inhibiting vertical mixing of heat and nutrients. Ship track current measurements noted strong alongshore NBC flow and a meandering NECC, which produces large-scale anticyclonic rings that are transported eastward. Nutrients, mainly ${\text{NO}}_{3}^{-}$ and ${\text{SiO}}_{2}^{-}$, were strongly depleted in coastal regions, and the autotrophy was greater than the heterotrophy (negative AOU). In terms of phytoplankton groups, diatoms dominated the region from the river mouth to the edge of the area affected by the NBC retroflection (Chl-*a* ranging from 0.02 to 0.94 mg m^−3^). Additionally, the NECC region is fully oligotrophic where the most representative groups are cyanobacteria and dinoflagellates (Chl-*a* ranging from 0.02 to 0.40 mg m^−3^). Copepods were the most diverse and abundant group of the zooplankton, playing a central role in the marine food web: 130 copepod species were identified, and they accounted for more than 60% of the zooplankton abundance. Two different zooplankton communities are represented in the area: a low-diversity, high-density coastal community present at inshore stations and a high-diversity, low-density oceanic community present at offshore stations. Copepods dominated offshore areas, whereas macrozooplankton (mainly Brachyura zoeae) dominated coastal stations under stronger plume influence. Based on the multivariate analysis, phytoplankton and zooplankton showed correlations with carbonate system parameters (DIC, TA, fCO_2_sw, HC${\text{NO}}_{3}^{-}$, C${\text{CO}}_{3}^{2-}$, and CO_2_ fluxes). The fCO_2_sw values reached 543 μatm in the coastal region but oscillated near the value of atmospheric fCO_2_ (379 μatm) offshore. Lower fCO_2_sw values were observed in the NECC area. The ΔfCO_2_ in this region was less than 5 μatm (−0.3 mmol CO_2_ m^−2^ d^−1^), while in the coastal region before retroflection, the ΔfCO_2_ value was approximately 50 μatm (+3.7 mmol CO_2_ m^−2^ d^−1^). The ΔfCO_2_ values varied considerably along the CF3 cruise, passing from high undersaturation in the coastal region (0.5°S) to oversaturation between 0° and 7°N. In the final portion of the ship track (45°–38°W), ΔfCO_2_ varied slightly between positive and negative values (8°N). Additionally, in the NECC region, blooms of species in the cyanophyte group (*Richelia sp*. and *Trichodesmium sp*.) were associated with the diatom group (*Rhizosolenia sp*.) and mesozooplankton (Copepods).

This study provides foundational data for future process-oriented high-resolution numerical modeling experiments, in which physical-biogeochemical mechanisms can be examined together as drivers of the observed geographical, seasonal and interannual variabilities in the AROC. These studies are currently underway.

## Author contributions

MA conceived the idea and coordinated the onboard activities during the CF3 cruise. MF and KT performed the chemical analysis. SN, RS, and PM performed the zooplankton analysis. AO and FF performed the phytoplankton analysis. MA, GH, and JA performed the physical analysis. CN, DV, NL, and LB performed the carbonate parameter analysis, CO_2_ analysis, and statistical tests. All authors contributed extensively to the interpretation of the results and to writing the manuscript.

### Conflict of interest statement

The authors declare that the research was conducted in the absence of any commercial or financial relationships that could be construed as a potential conflict of interest.

## References

[B1] Abril G. Martinez J.-M. Artigas L. F. Moreira-Turcq P. Benedetti M. F. Vidal L.. (2014). Amazon River carbon dioxide outgassing fuelled by wetlands. Nature 505, 395–398. 10.1038/nature12797 24336199

[B2] Araujo M. Noriega C. Lefèvre N. (2014). Nutrients and carbon fluxes in the estuaries of major rivers flowing into the tropical Atlantic. Front. Mar. 1:10 10.3389/fmars.2014.00010

[B3] Bates N. R. Hansell D. A. (2004). Temporal variability of excess nitrate and nitrogen fixation in the subtropical North Atlantic. Mar. Chem. 84, 225–241. 10.1016/j.marchem.2003.08.003

[B4] Baumgartner A. Reichel E. (1975). The World Water Balance. New York, NY: Elsevier.

[B5] Benson B. B. Krause D. (1984). The concentration and isotopic fractionation of oxygen dissolved in freshwater and seawater in equilibrium with the atmosphere. Limnol. Oceanogr. 29, 620–632.

[B6] Boltovskoy D. (1981). Atlas del zooplancton del Atlántico sudoccidental y metodos de trabajos con el zooplancton marino. Mar del Plata: INIDEP.

[B7] Boltovskoy D. (1999). South Atlantic Zooplankton. Leiden: Backhuys Publishers.

[B8] Bonou F. K. Noriega C. Lefèvre N. Araujo M. (2016). Distribution of CO_2_ parameters in the Western Tropical Atlantic Ocean. Dynam. Atmos. Ocean 73, 47–60. 10.1016/j.dynatmoce.2015.12.001

[B9] Brewer P. G. Goldman J. C. (1976). Alkalinity changes generated by phytoplankton growth. Limnol. Oceanogr. 21, 108–117.

[B10] Broecker W. S. Peng T. H. (1982). Tracers in the Sea. Palisades, NY: Eldigio Press.

[B11] Brzezinski M. A. (1985). The Si:C:N ratio of marine diatoms: Interspecific variability and the effect of some environmental variables. J. Phycol. 21, 347–357. 10.1111/j.0022-283646.1985.00347.x.

[B12] Carton J. A. (1991). Effect of seasonal surface freshwater flux on sea surface temperature in the Tropical Atlantic Ocean. J. Geophys. Res. 96, 12593–12598. 10.1029/91JC01256

[B13] Chen C.-T. A. Huang T.-H. Fu Y.-H. Bai Y. He X. (2012). Strong sources of CO_2_ in upper estuaries become sinks of CO_2_ in large river plumes. Curr. Opin. Environ. Sustainab. 4, 179–185. 10.1016/j.cosust.2012.02.003

[B14] Coles V. J. Brooks M. T. Hopkins J. Stukel M. R. Yager P. L. Hood R. R. (2013). The pathways and properties of the Amazon River plume in the tropical North Atlantic Ocean. J. Geophys. Res. Oceans. 118, 6894–6913. 10.1002/2013JC008981

[B15] Conroy B. (2016). Zooplankton Community Composition and Grazing in the Amazon River plume and Western Tropical North Atlantic Ocean. Dissertations, Theses, and Masters Projects. Paper 1477068157, College of William and Mary - Virginia Institute of Marine Science.

[B16] Cooley S. R. Coles V. J. Subramaniam A. Yager P. L. (2007). Seasonal variations in the Amazon plume-related atmospheric carbon sink. Global Biogeochem. Cycles 21:GB3014 10.1029/2006GB002831

[B17] Cooley S. R. Yager P. L. (2006). Physical and biological contributions to the western tropical North Atlantic Ocean carbon sink formed by the Amazon River plume. J. Geophys. Res. 111:C08018 10.1029/2005JC002954

[B18] da Cunha L. C. Buitenhuis E. T. (2013). Riverine influence on the tropical Atlantic Ocean biogeochemistry. Biogeosciences 10, 6357–6373. 10.5194/bg-10-6357-2013

[B19] DeMaster D. Aller R. (2001). Biogeochemical processes on the Amazon shelf: changes in dissolved and particulate fluxes during river/ocean mixing, in The Biogeochemistry of the Amazon Basin, eds Mc Clain M. Victoria R. Richey J. (New York, NY: Oxford University Press), 328–357.

[B20] DeMaster D. J. Pope R. H. (1996). Nutrient dynamics in Amazon shelf waters: results from Amassed. Cont Shelf Res. 16, 263–289.

[B21] DeMaster D. J. Smith W. O. Jr. Nelson D. M. Aller J. Y. (1996). Biogeochemical processes in Amazon shelf waters: chemical distributions and uptake rates of silicon, carbon and nitrogen. Cont. Shelf Res. 16, 617–643.

[B22] Detoni A. M. S. Ciotti Á. M. Calil P. H. R. Tavano V. M. Yunes J. S. (2016). *Trichodesmium* latitudinal distribution on the shelf break in the southwestern Atlantic Ocean during spring and autumn. Global Biogeochem. Cycles 30, 1738–1753. 10.1002/2016GB005431

[B23] Deutsch C. Gruber N. Key R. M. Sarmiento J. L. (2001). Denitrification and N2 fixation in the Pacific Ocean. Global Biogeochem. Cycles. 15, 483–506. 10.1029/2000GB001291

[B24] Dickson A. G. (1990a). Standard potential of the reaction : AgCl(s) + 1/2H_2_(g) = Ag(s) + HCl(aq), and the standard acidity constant of the ion HSO_4_ in synthetic sea water from 273.15 to 318.15 K. J. Chem. Thermodyn. 22, 113–127. 10.1016/0021-9614(90)90074-Z

[B25] Dickson A. G. (1990b). Thermodynamics of the dissociation of boric acid in synthetic seawater from 273.15 to 318.15 K. Deep Sea Res. I Oceanogr. Res. Papers 37, 755–766. 10.1016/0198-0149(90)90004-F

[B26] Dickson A. G. Millero F. J. (1987). A comparison of the equilibrium constants for the dissociation of carbonic acid in seawater media. Deep Sea Res. 34, 1733–1743.

[B27] Edmond J. M. (1970). High precision determination of titration alkalinity and total carbon dioxide content of sea water by potentiometric titration. Deep Sea Res. Oceanogr. 17, 737–750. 10.1016/0011-7471(70)90038-0

[B28] Edmond J. M. Boyle E. A. Grant B. Stallard R. F. (1981). The chemical mass balance in the Amazon plume I: The nutrients. Deep Sea Res. Part A Oceanogr. Res. 28a, 1339–1374.

[B29] Ffield A. (2005). North Brazil current rings viewed by TRMM Microwave Imager SST and the influence of the Amazon Plume. Deep Sea Res. Part I 52, 137–160. 10.1016/j.dsr.2004.05.013

[B30] Fonseca C. A. Goni G. J. Johns W. E. Campos E. J. D. (2004). Investigation of the North Brazil Current retroflection and North Equatorial Countercurrent variability. Geophys. Res. Lett. 31:L21304 10.1029/2004GL020054

[B31] Garcia F. H. Gordon I. I. (1992). Oxygen solubility in seawater: better fitting equations. Limnol. Oceanogr. 37, 1307–1312.

[B32] Garcia H. E. Locarnini R. A. Boyer T. P. Antonov J. I. (2006). World Ocean Atlas 2005, Volume 3: Dissolved Oxygen, Apparent Oxygen Utilization, and Oxygen Saturation. ed Levitus S. (Washington, DC: NOAA Atlas NESDIS; U.S. Government Printing Office).

[B33] Grasshoff K. Ehrhardt M. Kremling K. (1983). Methods of Seawater Analysis, 2nd Edn. New York, NY: Verlag Chemie.

[B34] Gruber N. Sarmiento J. (1997). Global patterns of marine nitrogen fixation and denitrification. Global Biochem. Cycles 11, 235–266.

[B35] Guiry M. D. Guiry G. M. (2016). AlgaeBase. World-wide Electronic Publication. Galway: National University of Ireland Available online at: http://www.algaebase.org (Accessed: 24 july 2016)

[B36] Hansell D. Follow M. (2008). Nitrogen in the Atlantic Ocean, in Nitrogen in the Marine Environment, eds. Capone D. G. Bronk D. Mulholland M. R. Carpenter E. J. (Amsterdam: Academic Press), 597–630.

[B37] Hansell D. A. Bates N. R. Olson D. B. (2004). Excess nitrate and nitrogen fixation in the North Atlantic. Mar. Chem. 284, 243–265. 10.1016/j.marchem.2003.08.004

[B38] Huffman G. J. Adler R. Bolvin D. Gu G. Nelkin E. Bowman K. (2007). The TRMM Multisatellite Precipitation Analysis (TMPA): quasi-global, multiyear, combined-sensor precipitation estimates at fine scales. J. Hydrometeorol. 8, 38–55. 10.1175/JHM560.1

[B39] Ibánhez J. S. P. Araujo M. Lefèvre N. (2016). The overlooked tropical oceanic CO_2_ sink. Geophys. Res. Lett. 43, 3804–3812. 10.1002/2016GL068020

[B40] Ibánhez J. S. P. Diverrès D. Araujo M. Lefèvre N. (2015). Seasonal and interannual variability of sea-air CO_2_ fluxes in the tropical Atlantic affected by the Amazon River plume. Global Biogeochem. Cycles 29, 1640–1655. 10.1002/2015GB005110

[B41] Kitidis V. Hardman-Mountford N. J. Litt E. Brown I. Cummings D. Hartman S. (2012). Seasonal dynamics of the carbonate system in the Western English Channel. Cont. Shelf Res. 42, 30–40. 10.1016/j.csr.2012.04.012

[B42] Koffi U. Lefèvre N. Kouadio G. Boutin J. (2010). Surface CO_2_ parameters and air–sea CO_2_ fluxes distribution in the eastern equatorial Atlantic Ocean. J. Marine Sys. 82, 135–144. 10.1016/j.jmarsys.2010.04.010

[B43] Körtzinger A. (2003). A significant CO_2_ sink in the tropical Atlantic Ocean associated with the Amazon River plume. Geophys. Res. Lett. 30:2287 10.1029/2003GL018841

[B44] Lefèvre N. Diverrès D. Gallois F. (2010). Origin of CO_2_ undersaturation in the western tropical Atlantic. Tellus B. 62, 595–607. 10.1111/j.1600-0889.2010.00475.x

[B45] Lefèvre N. Moore G. Aiken J. Watson A. Cooper D. Ling R. (1998). Variability of pCO_2_ in the tropical Atlantic in 1995. J. Geophys. Res. 103, 5623–5634. 10.1029/97JC02303

[B46] Lefevre N. Urbano D. F. Gallois F. Diverres D. Lefèvre N. Urbano D. F. (2014). Impact of physical processes on the seasonal distribution of the fugacity of CO_2_ in the western tropical Atlantic. J. Geophys. Res. Ocean. 119, 646–663. 10.1002/2013JC009248

[B47] Lobo E. Leighton G. (1986). Estructuras comunitárias de las fitocenosia planctonicas de los sistemas de desembocaduras de rios y esteros de Ia zona central de Chile. Rev. Biol,. Marina 22, 1–29.

[B48] Luo Y.-W. Doney S. C. Anderson L. A. Benavides M. Berman-Frank I. Bode A. (2012). Database of diazotrophs in global ocean: abundance, biomass and nitrogen fixation rates. Earth Syst. Sci. Data 4, 47–73. 10.5194/essd-4-47-2012

[B49] Mehrbach C. Culberson C. H. Hawley J. E. Pytkowicz R. M. (1973). Measurement of the apparent dissociation constants of carbonic acid in seawater at atmospheric pressure. Limnol. Oceanogr. 18, 897–907. 10.4319/lo.1973.18.6.0897

[B50] Newell G. H. Newell R. (1963). Marine Plankton: A Practical Guide. London: Hutchinson Education.

[B51] Noriega C. E. D. Araujo M. Lefèvre N. (2013). Spatial and Temporal Variability of the CO_2_ Fluxes in a Tropical, Highly Urbanized Estuary. Estuar. Coasts 36, 1054–1072. 10.1007/s12237-013-9608-1

[B52] Omori M. Ikeda T. (1984). Methods of Marine Zooplankton Ecology. New York, NY: John Wiley.

[B53] Pielou E. C. (1977). Mathematical Ecology. New York, NY: John Wiley & Sons.

[B54] Richardson P. L. Reverdin G. (1987). Seasonal cycle of velocity in the Atlantic North Equatorial Countercurrent as measured by surface drifters, current meters, and ship drifts. J. Geophys. Res. 92, 3691–3708. 10.1029/JC092iC04p03691

[B55] Richey J. E. Melack J. M. Aufdenkampe A. K. Ballester V. M. Hess L. L. (2002). Outgassing from Amazonian rivers and wetlands as a large tropical source of atmospheric CO_2_. Nature 416, 617–620. 10.1038/416617a 11948346

[B56] Richey J. E. Nobre C. Desser C. (1989). Amazon River discharge and climate variability: 1903–1985. Science 246, 101–103. 10.1126/science.246.4926.10 17837767

[B57] Richey J. Hedges J. Devol A. Quay P. Victoria R. Martinelli L. (1990). Biogeochemistry of carbon in the Amazon River. Limnol Oceanogr. 35, 352–371.

[B58] Robbins L. Hansen M. Kleypas J. Meylan S. (2010). CO2calc: A User-Friendly Seawater Carbon Calculator for Windows, Mac OS X, and iOS (iPhone). 17. Available online at: http://www.usgs.gov/pubprod

[B59] Shannon C. E. (1948). A mathematical theory of communication. AT T Tech J. 27, 379–423.

[B60] Shipe R. F. Carpenter E. J. Govil S. R. Capone D. G. (2007). Limitation of phytoplankton production by Si and N in the western Atlantic Ocean. Mar. Ecol. Prog. Ser. 338, 33–45. 10.3354/meps338033

[B61] Silva A. C. Araujo M. Bourlès B. (2010). Seasonal variability of the Amazon River plume during REVIZEE Program. Trop. Oceanogr. 38, 70–81. 10.5914/tropocean.v38i1.5162

[B62] Silva A. C. Bourlès B. Araujo M. (2009). Circulation of the thermocline salinity maximum waters off the Northern Brazil as inferred from *in situ* measurements and numerical results. Annales Geophysicae. 27, 1861–1873. 10.5194/angeo-27-1861-2009

[B63] Silva A. C. M. Araujo C. Medeiros M. Silva B. Bourlès (2005). Seasonal changes in the mixed and barrier layers in the western equatorial Atlantic. Brazilian J. Oceanogr. 53, 83–98. 10.1590/S1679-87592005000200001

[B64] Skirrow G. (1975). The dissolved gases–carbon dioxide, in Chemical Oceanography, Vol. 2, eds Riley Z. J. P. Skirrow G. (London: Academic Press), 1–192.

[B65] Smith W. O. Jr. Demaster D. J. (1996). Phytoplankton biomass and productivity in the Amazon River plume: correlation with seasonal river discharge. Cont. Shelf Res. 16, 291–319. 10.1016/0278-4343(95)00007-N

[B66] Stramma L. Schott F. (1999). The mean flow field of the tropical Atlantic Ocean. Deep Sea Res. II. 46, 279–303.

[B67] Strickland J. D. H. Parsons T. R. (1972). A Pratical Handbook of Seawater Analysis, 2nd Edn. Ottawa, ON: Fisheries Research Board of Canada Bulletim.

[B68] Subramaniam A. Yager P. L. Carpenter E. J. Mahaffey C. Bjorkman K. Cooley S.. (2008). Amazon River enhances diazotrophy and carbon sequestration in the tropical North Atlantic Ocean. Proc. Natl. Acad. Sci. U.S.A. 105, 10460–10465. 10.1073/pnas.0710279105 18647838 PMC2480616

[B69] Sweeney C. Gloor E. Jacobson A. R. Key R. M. McKinley G. Sarmiento J. L. (2007). Constraining global air-sea gas exchange for CO_2_ with recent bomb 14C measurements. Global Biogeochem. Cycles 21, 1–10. 10.1029/2006GB002784

[B70] Ternon J. F. Oudot C. Dessiera A. Diverres D. (2000). A seasonal tropical sink for atmospheric CO_2_ in the Atlantic ocean: the role of the Amazon River discharge. Mar. Chem. 68, 183–201. 10.1016/S0304-4203(99)00077-8

[B71] The Department of Energy (1994). Handbook of Methods for the Analysis of the Various Parameters of the Carbon Dioxide System in Sea Water. Version 2, edited by Dickson A. G. Goyet C. ORNL/CDIAC-74. Available online at: http://cdiac.ornl.gov/oceans/handbook.html

[B72] UNESCO (1966). Determination of Photosynthetic Pigments in Seawater. Paris: Imprimerie Rolland-Paris.

[B73] Urbano D. F. De Almeida R. A. F. Nobre P. (2008). Equatorial undercurrent and North equatorial countercurrent at 38°W: a new perspective from direct velocity data. J. Geophys. Res. Ocean. 113, 1–16. 10.1029/2007JC004215

[B74] Weiss R. F. (1974). Carbon dioxide in water and seawater: the solubility of a non-ideal gas. Mar. Chem. 2, 203–215. 10.1016/0304-4203(74)90015-2

[B75] Wilson W. D. Johns W. E. Garzoli S. L. (2002). Velocity structure of the North Brazil Current rings. Geophys Res Lett. 29, 114-1–114-3. 10.1029/2001GL013869

[B76] Yeung L. Y. Berelson W. M. Young E. D. Prokopenko M. G. Rollins N. Coles V. J. (2012). Impact of diatom-diazotroph associations on carbon export in the Amazon River plume. Geophys. Res. Lett. 39:L18609 10.1029/2012GL053356.

[B77] Yoo J. M. Carton J. A. (1990). Annual and interannual variations of the freshwater budget in the Tropical Atlantic Ocean and the Caribbean Sea. J. Phys. Oceanogr. 20, 831–845.

[B78] Yu L. Jin X. Weller R. (2008). Multidecade Global Flux Datasets from the Objectively Analyzed Air-sea Fluxes (OAFlux) Project: Latent and Sensible Heat Fluxes, Ocean Evaporation, and Related Surface Meteorological Variables. Woods Hole Oceanographic Institution OAFlux Project Technical Report (OA-2008-01).

[B79] Zar J. H. (1996). Biostatistical Analysis, 1st Edn. Prentice Hall.

[B80] Zeebe R. E. Wolf-Gladrow D. (2001). CO_2_ in Seawater: Equilibrium, Kinetics, Isotopes, Vol. 65, 1st Edn. Amsterdam: Elsevier Science.

