# Supplementary material for: A Synoptic Assessment of the Amazon River-Ocean Continuum during Boreal Autumn: From Physics to Plankton Communities and Carbon Flux
Source: Front Microbiol. 2017 Jul 31;8:1358. doi: 10.3389/fmicb.2017.01358 (PMC5534475; doi:10.3389/fmicb.2017.01358)
Supplement: Supplementary file 1 [file Image3.PDF]

Supplementary material

A synoptic assessment of the Amazon River-Ocean Continuum during boreal autumn: from physics to plankton communities and carbon flux

Moacyr Araujo[1,2,*], Carlos Noriega[1,2], Gbekpo Hounsou-gbo[1,2,3], Doris Veleda[1,2], Julia Araujo[1], Leonardo Bruto[1,2], Fernando Feitosa[1], Manuel Flores-Montes[1], Nathalie Lefèvre[4], Pedro Melo[1], Amanda Otsuka[1], Keyla Travassos, Ralf Schwamborn[1], Sigrid Neumann-Leitão[1].

[1] Department of Oceanography – DOCEAN, Federal University of Pernambuco – UFPE, Av. Arquitetura s/n, 50740-550, Recife-PE, Brazil.
[2] Brazilian Research Network on Global Climate Change – Rede CLIMA, Av. dos Astronautas 1758, 12227-010, São José dos Campos-SP, Brazil.
[3] International Chair in Mathematical Physics and Applications (ICMPA-Unesco Chair), UAC, Cotonou, Bénin.
[4] IRD-LOCEAN, Sorbonne Universités (Université Pierre et Marie Curie CNRS-MNHN), 4 place Jussieu, 75252 Paris Cedex 05, France.

1. Removal (%) method

The mixing lines for DIN, PO_4^- and SiO_2^- were constructed from Amazon River estuary surface samples (Key et al., 1985; Fox et al. 1986; Ternon et al. 2000; Araujo et al. 2014) and from mean oceanic concentration determined during our cruises, resulting in the following endmembers (Figure 1S):

Ocean water: Salinity>34.9; NO3-=11 μmol l^{-1}; PO_4^-=0.1 μmol l^{-1} and SiO_2^-=1.1 μmol l^{-1}.

River water: Salinity=0; NO3-=16 μmol l^{-1}; PO_4^-=1 μmol l^{-1} and SiO_2^-=142 μmol l^{-1}.

The removals of NO3-, PO_4^- and SiO_2^- in the N region (Amazon River plume) were calculated according to Noriega et al. (2013):

$$\mathrm{Removal}(\%) = \frac{c_0 - c^*}{c_0} \times 100 \quad (1S)$$

where c_0 is the NO3-, PO_4^- or SiO_2^- concentration at zero salinity, and c* is the effective concentration in each station.

Figures

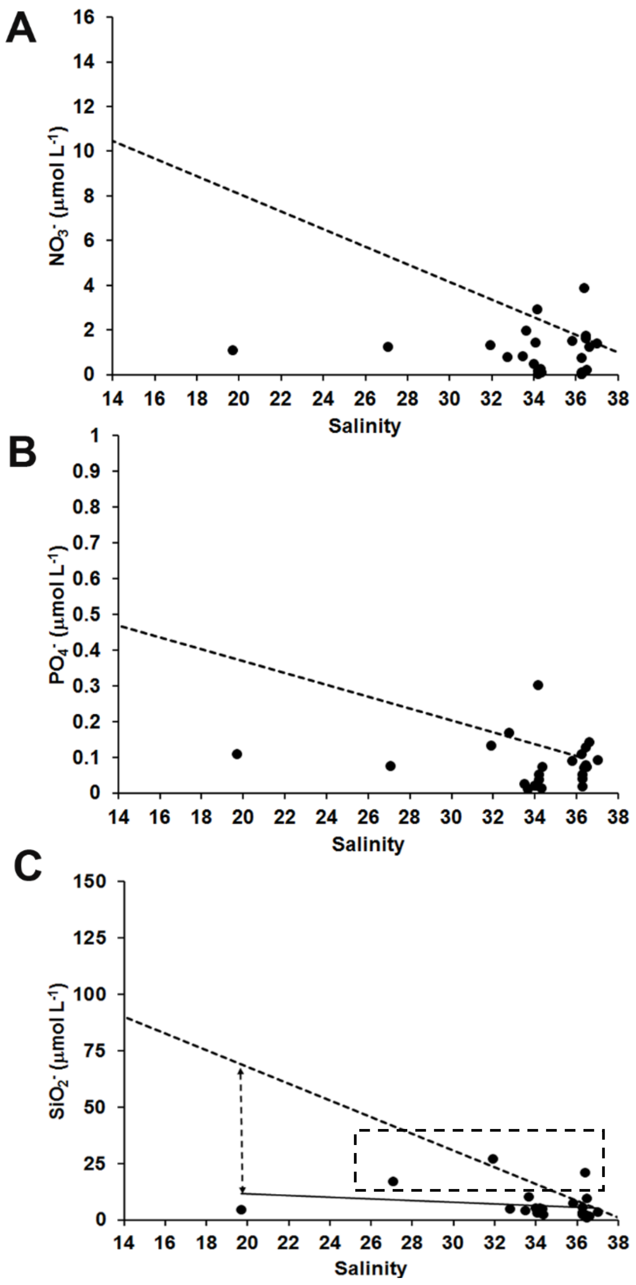

Figure 1S. Plots of NO_3^-, PO_4^-, and SiO_2^- vs salinity (a, b and c, respectively). The dashed line represents the conservative mixing between river and sea waters, whose endmembers are defined in the text. The solid line represents the mixing line obtained in CF3 cruise for SiO_2^-. Vertical dashed line in (c) indicate maximum depression of SiO_2^-. The box indicates where the greatest amount of diatoms occurred (mainly *Fragillaria sp*. and *Pseudo-nitzschia pungens*).

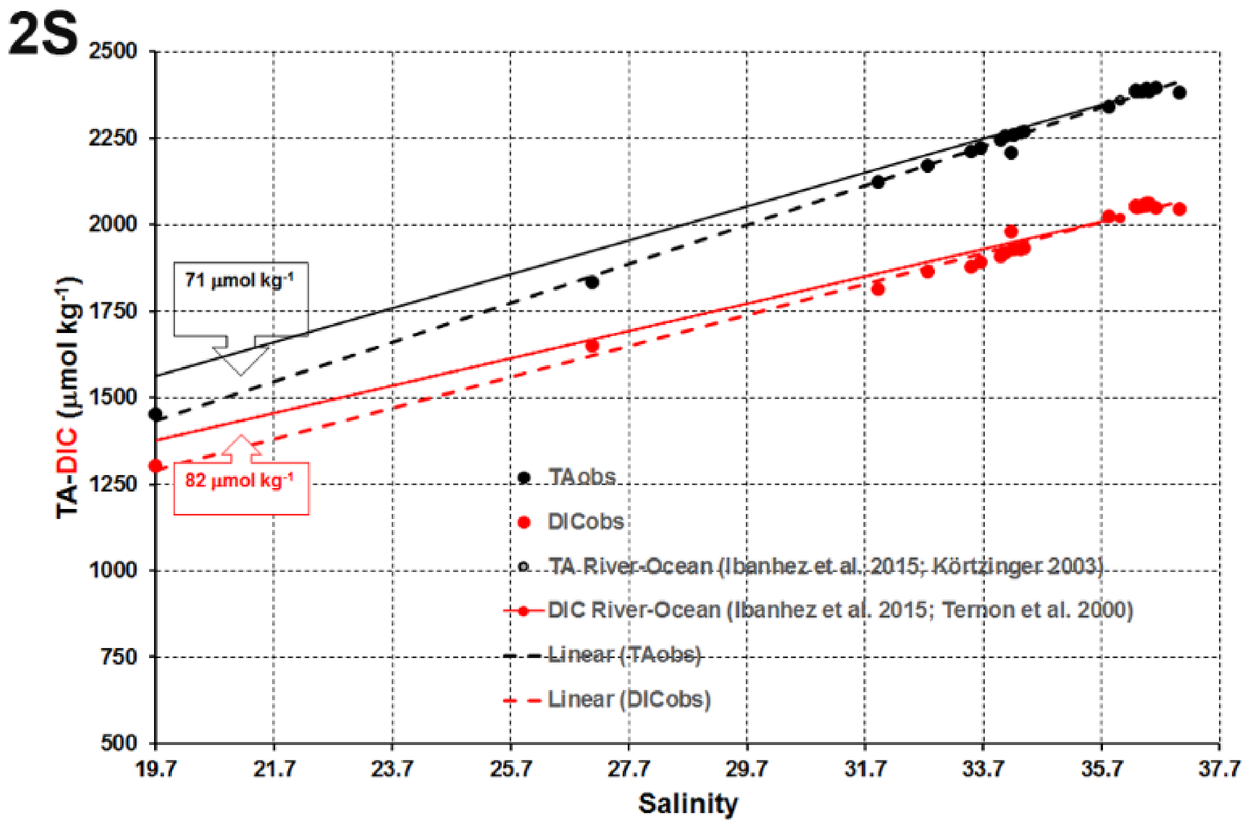

Figure 2S. Plot of TA and DIC vs salinity. The solid line represents the conservative mixing between river and sea waters, whose endmembers are defined in the text. The dashed line represents the mixing line obtained in CF3 cruise.

References

Araujo, M., Noriega, C. and Lefèvre, N., 2014. Nutrients and carbon fluxes in the estuaries of major rivers flowing into the tropical Atlantic. Front. Mar. 1(10), 1-16. doi: 10.3389/fmars.2014.00010.

Fox, L.E., Sager, S.L., Wofsy, S.C., 1986. The chemical control of soluble phosphorus in the Amazon estuary. Geochim. Cosmochim. Acta 50, 783–794.

Key, R.M., Stallard, R.F., Moore, W.S., Sarmiento, J.L., 1985. Distribution of Ra-226 and Ra-228 in the Amazon River estuary. J. Geophys. Res. 90, 6905-7004.

Noriega, C.E.D., Araujo, M., Lefèvre, N., 2013. Spatial and Temporal Variability of the CO2 Fluxes in a Tropical, Highly Urbanized Estuary. Estuaries and Coasts 36, 1054–1072.

Ternon, J.F., Oudot, C., Dessier, A., Diverres, D., 2000. A seasonal tropical sink for atmospheric CO_2 in the Atlantic Ocean: the role of the Amazon River discharge. Mar. Chem. 68, 183–201.
